# Decreasing-Rate Pruning Optimizes the Construction of Efficient and Robust Distributed Networks

**DOI:** 10.1371/journal.pcbi.1004347

**Published:** 2015-07-28

**Authors:** Saket Navlakha, Alison L. Barth, Ziv Bar-Joseph

**Affiliations:** 1 Center for Integrative Biology, The Salk Institute for Biological Studies, La Jolla, California, United States of America; 2 Department of Biological Sciences, Center for the Neural Basis of Cognition, Carnegie Mellon University, Pittsburgh, Pennsylvania, United States of America; 3 Lane Center for Computational Biology, Machine Learning Department, Carnegie Mellon University, Pittsburgh, Pennsylvania, United States of America; Université Paris Descartes, Centre National de la Recherche Scientifique, FRANCE

## Abstract

Robust, efficient, and low-cost networks are advantageous in both biological and engineered systems. During neural network development in the brain, synapses are massively over-produced and then pruned-back over time. This strategy is not commonly used when designing engineered networks, since adding connections that will soon be removed is considered wasteful. Here, we show that for large distributed routing networks, network function is markedly enhanced by hyper-connectivity followed by aggressive pruning and that the global rate of pruning, a developmental parameter not previously studied by experimentalists, plays a critical role in optimizing network structure. We first used high-throughput image analysis techniques to quantify the rate of pruning in the mammalian neocortex across a broad developmental time window and found that the rate is decreasing over time. Based on these results, we analyzed a model of computational routing networks and show using both theoretical analysis and simulations that decreasing rates lead to more robust and efficient networks compared to other rates. We also present an application of this strategy to improve the distributed design of airline networks. Thus, inspiration from neural network formation suggests effective ways to design distributed networks across several domains.

## Author Summary

During development of neural circuits in the brain, synapses are massively over-produced and then pruned-back over time. This is a fundamental process that occurs in many brain regions and organisms, yet, despite decades of study of this process, the rate of synapse elimination, and how such rates affect the function and structure of networks, has not been studied. We performed large-scale brain imaging experiments to quantify synapse elimination rates in the developing mouse cortex and found that the rate is decreasing over time (i.e. aggressive elimination occurs early, followed by a longer phase of slow elimination). We show that such rates optimize the efficiency and robustness of distributed routing networks under several models. We also present an application of this strategy to improve the design of airline networks.

## Introduction

Neural networks in the brain are formed during development using a pruning process that includes expansive growth of synapses followed by activity-dependent elimination. In humans, synaptic density peaks around age 2 and subsequently declines by 50–60% in adulthood [1–4]. It has been hypothesized that synaptic pruning is important for experience-dependent selection of the most appropriate subset of connections [1, 5], and it occurs in many brain regions and species [6–9]. This strategy substantially reduces the amount of genetic information required to code for the trillions of connections made in the human brain [10]. Instead of instructing precise connections, more general rules can be applied, which are then fine-tuned by activity-dependent selection. Although the molecular and cellular mechanisms driving activity-dependent pruning have been extensively investigated [1, 3, 4], global aspects of this highly-distributed process, including the rate at which synapses are pruned, the impact of these rates on network function, and the contrast of pruning-versus growth-based strategies commonly used in engineering to construct networks, has not been studied.

While the specific computations performed within neural and engineered networks may be very different, at a broad level, both types of networks share many goals and constraints [11]. First, networks must propagate signals efficiently while also being robust to malfunctions (e.g. spike propagation failures in neural networks [12–14]; computer or link failures in communication networks [15]). Second, both types of networks must adapt connections based on patterns of input activity [16]. Third, these factors must be optimized under the constraint of distributed processing (without a centralized coordinator) [17, 18], and using low-cost solutions that conserve important metabolic or physical resources (e.g. number of synapses or wiring length in biological networks; energy consumption or battery-life in engineered networks) [19–21]. For example, on the Internet or power grid, requests can be highly dynamic and variable over many time-scales and can lead to network congestion and failures if networks are unable to adapt to such conditions [22, 23]. In wireless or mobile networks, broadcast ranges (which determine network topology) need to be inferred in real-time based on the physical distribution of devices in order to optimize energy efficiency [24]. Although optimizing network design is critical for such engineered systems across a wide range of applications, existing algorithms used for this problem are not, to our knowledge, based on experience-based pruning, in part because adding connections that will soon be eliminated is considered wasteful.

Here, we develop a computational approach informed by experimental data to show that pruning-inspired algorithms can enhance the design of distributed routing networks. First, we experimentally examined developmental pruning rates in the mouse somatosensory cortex, a well-characterized anatomical structure in the mouse brain [25]. Using electron microscopy imaging across 41 animals and 16 developmental time-points, coupled with unbiased and high-throughput image analysis [26], we counted over 20,000 synapses and determined that pruning rates are decreasing over time (i.e. early, rapid synapse elimination is followed by a period of slower, gradual elimination). Next, to translate these observations to the computational domain, we developed a simulated environment for comparing algorithms for distributed network construction. We find that over-connection followed by pruning leads to significant improvements in efficiency (routing distance in the network) and robustness (number of alternative routes between two nodes) compared to commonly-used methods that add connections to initially-sparse networks. To determine if these results hold more generally, we analyzed the theoretical basis of network construction by pruning and found that decreasing rates led to networks with near-optimal connectivity compared to other rates (increasing, constant, etc.), which we also confirmed using simulations. Finally, we adapted a pruning-based strategy to improve the design of airline networks using real traffic pattern data.

The novelty of our approach is two-fold. First, while synaptic pruning has been studied for decades, previous analyses have determined that synaptic density peaks during early development and is reduced by late adolescence and adulthood [6–9]. However, fine-scale measurements to statistically establish the rate of synapse elimination have not been made. Second, while substantial prior work linking neural and computational networks has focused on the *computation* performed by neural networks [27, 28], our work focuses on the *construction* of networks and provides a quantitative platform to compare different network construction processes based on their cost, efficiency, and robustness. Our goals here are to model pruning from an abstract, graph-theoretic perspective; we do not intend to capture all the requirements of information processing in the brain, and instead focus on using pruning-inspired algorithms for improving routing in distributed networks. Overall, our results suggest that computational thinking can simultaneously lead to novel, testable biological hypotheses and new distributed computing algorithms for designing better networks.

## Results

### Neural networks employ decreasing rates of synapse elimination

Many generative models have been proposed to understand how networks evolve and develop over time (e.g. preferential attachment [29], small-world models [30], duplication-divergence [31, 32]), yet most of these models assume that the number of nodes and edges strictly grows over time. Synaptic pruning, however, diverges from this strategy. To better understand how pruning is implemented and whether it can be used to construct networks for broad routing problems, we sought to measure this process experimentally. Although pruning is a well-established neurodevelopmental phenomenon, previous experimental studies have primarily focused on identifying the time period over which pruning begins and ends but have largely ignored the dynamics in between these end-points [6, 9, 33], lacking crucial pruning rate information that may be useful for using pruning-based strategies for building distributed networks.

**Fig 1:**
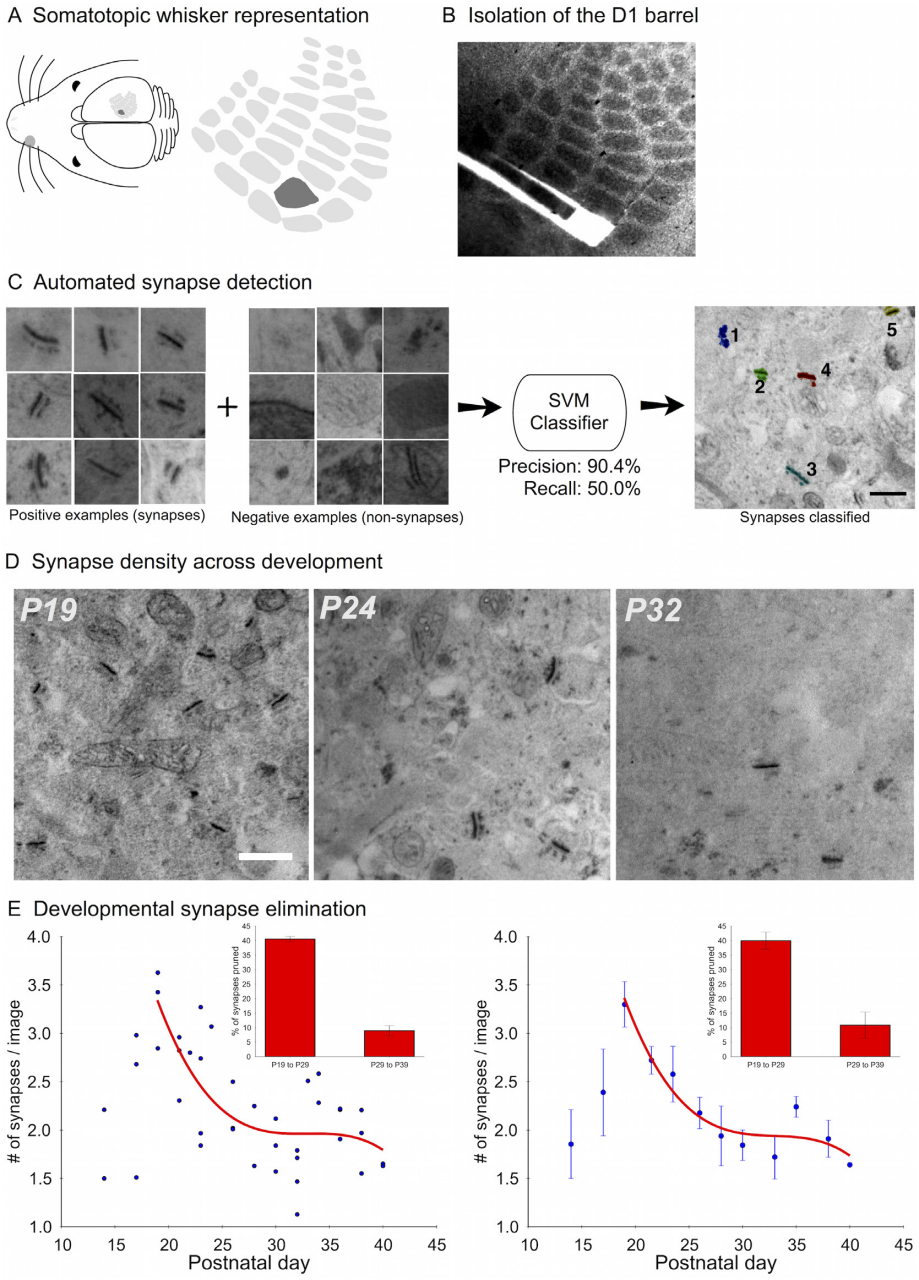
Experimental pipeline, image processing, and decreasing pruning rates. (A) Schematic of the somatotopic mapping of whiskers to neocortical columns in the mouse somatosensory cortex. (B) Tangential sections from flattened brains preserve the structure of the barrel-field, enabling easy identification and isolation of the D1 barrel in tissue from different ages. (C) A support vector machine (SVM) classifier is trained using manually labeled examples of synapses and non-synapses in electron microscopy images. (D) Example images of synapses at three different time points corresponding to peak synapse density (P19), and later drop-off (P24 and P32). Scale bar represents 500nm. (E) Developmental pruning rate (raw data, left; binned data, right). Red lines show spline interpolations of the data points. Insets show that the majority of synapses are pruned during the first half of the developmental pruning period.

To determine the rate of synapse loss in developing neural networks, we focused on a well-characterized region of the neocortex, layer 4 of somatosensory cortex representing the D1 whisker (Fig 1A), where both thalamic inputs and recurrent circuitry are established in the first two postnatal weeks [34–36]. Because this region of primary sensory cortex does not receive significant input from other cortical layers [37], measurements of synaptic pruning reflect the maturation of an extant network, uncontaminated by the addition of synapses over the analysis window. In addition, the somatotopic anatomy of the whisker (barrel) cortex insured that comparisons across different animals and time-points could be made for the identical small cortical region (Fig 1B).

Changes in synaptic density over time were obtained from sampling 41 animals over 16 developmental time-points ranging from postnatal day 14 (P14) to P40 (Table 1). Over 20,000 synapses in nearly 10,000 images were identified using a synapse-enhancing reaction that specifically highlights synaptic contacts for electron microscopy [38, 39], coupled with unbiased machine learning algorithms (Fig 1C; Materials and Methods) [26]. Consistent with prior estimates that sampled only the peak and the end-point [9, 33], peak synaptic density occurred at P19 and density declined steeply to mature levels three weeks later (Fig 1D and 1E). Synapse density at P40 was similar to adult mice sampled at P65 (S4 Fig).

**Table 1:** Overview of experiments and synapses detected.

**Post-natal day (P)**	**# Animals**	**# Images**	**# Synapses (Avg/Image)**	**Precision**	**Recall**
14	2	269	500 (1.86)	89.00	50.00
17	3	400	964 (2.41)	92.40	50.00
19	3	739	2437 (3.30)	88.37	49.50
21	3	736	1984 (2.70)	96.57	49.70
22	2	397	1121 (2.83)	80.80	50.00
23	4	767	1913 (2.50)	92.75	49.88
24	1	136	418 (3.07)	96.50	49.50
26	3	523	1176 (2.25)	91.93	49.83
28	2	915	1779 (1.95)	95.70	49.45
30	3	642	1186 (1.85)	95.40	49.83
32	4	1480	2192 (1.48)	96.00	49.70
33	1	256	642 (2.51)	98.00	50.00
34	2	508	1235 (2.43)	88.80	49.50
36	3	763	1611 (2.11)	90.83	49.73
38	3	734	1397 (1.91)	92.63	49.80
40	2	489	803 (1.64)	94.10	49.65
16	41	9754	21355	92.38	49.76

**Fig 2:**
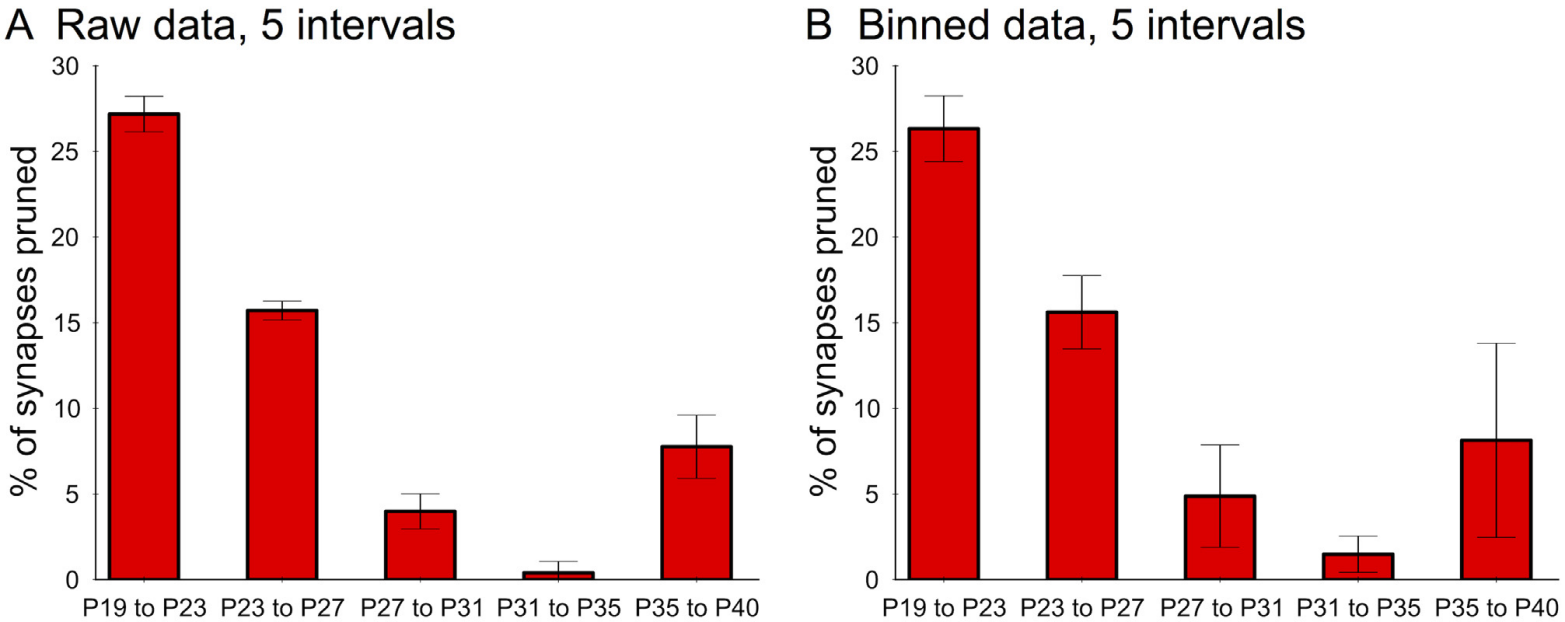
Finer analysis of decreasing synaptic pruning rates. The pruning period was divided into 5 intervals and the percentage of synapses pruned across successive intervals is depicted by the red bars. Statistics were computed using a leave-out-one strategy on either individual samples from the raw data (A) or on entire time-points using the binned data (B), where samples from a 2-day window were merged into the same time-point. Error bars indicate standard deviation over the cross-validation folds. All successive points are significantly different (*P* < 0.01, two-sample t-test).

Pruning rates were decreasing over time, i.e. rapid elimination was followed by a slower period of pruning. To determine the significance of this observation and to test whether only a single sample or time-point was driving the rate, we used a leave-one-out cross-validation strategy (Materials and Methods). First, the pruning period was divided into either 2 or 5 equally-spaced intervals over time from P19 to P40. Second, for each fold in the cross-validation, either one sample was left-out or one time-point was left-out. Third, a spline interpolation curve was fit and was used to compute the percentage of synapses pruned across successive intervals. When dividing the period into 2 intervals (P19–P29, *n* = 18 animals and P29–P39, *n* = 18 animals), there was a significant decrease in the percentage of synapses pruned within the first interval compared to the second interval (average percentage of synapses pruned from P19 to P29: 39.99%; (standard deviation over cross-validation folds: 2.93); average percentage of synapses pruned from P29 to P39: 10.87% (standard deviation: 4.56); *P* < 0.01, unpaired 2-sample t-test; Fig 1E). When dividing into 5 intervals, we also found a significant decrease in percentage of synapses pruned within the first interval versus the second (27% versus 15%; *P* < 0.01 unpaired 2-sample t-test) and similar decreases across the next two intervals (Fig 2). The slight rise in pruning in the last interval (7%) may be due to the addition of layer-4-innervating afferents from other brain areas [40] (indeed, we see a small rise in synapse density at P33, followed by additional pruning; S6 Fig). Nonetheless, the majority of the pruning still occurs during the first two intervals compared to the last three (*P* < 0.01), which is quantitatively indicative of a decreasing rate.

To further assess the reproducibility of these results, synapse density was adjusted for 3D analysis, which also confirmed a decreasing rate of synapse elimination (S5 Fig). These data indicate that neural networks are modified by aggressive pruning of connections, followed by a later, slow phase of synaptic elimination.

### Pruning outperforms growing algorithms for constructing distributed networks

**Fig 3:**
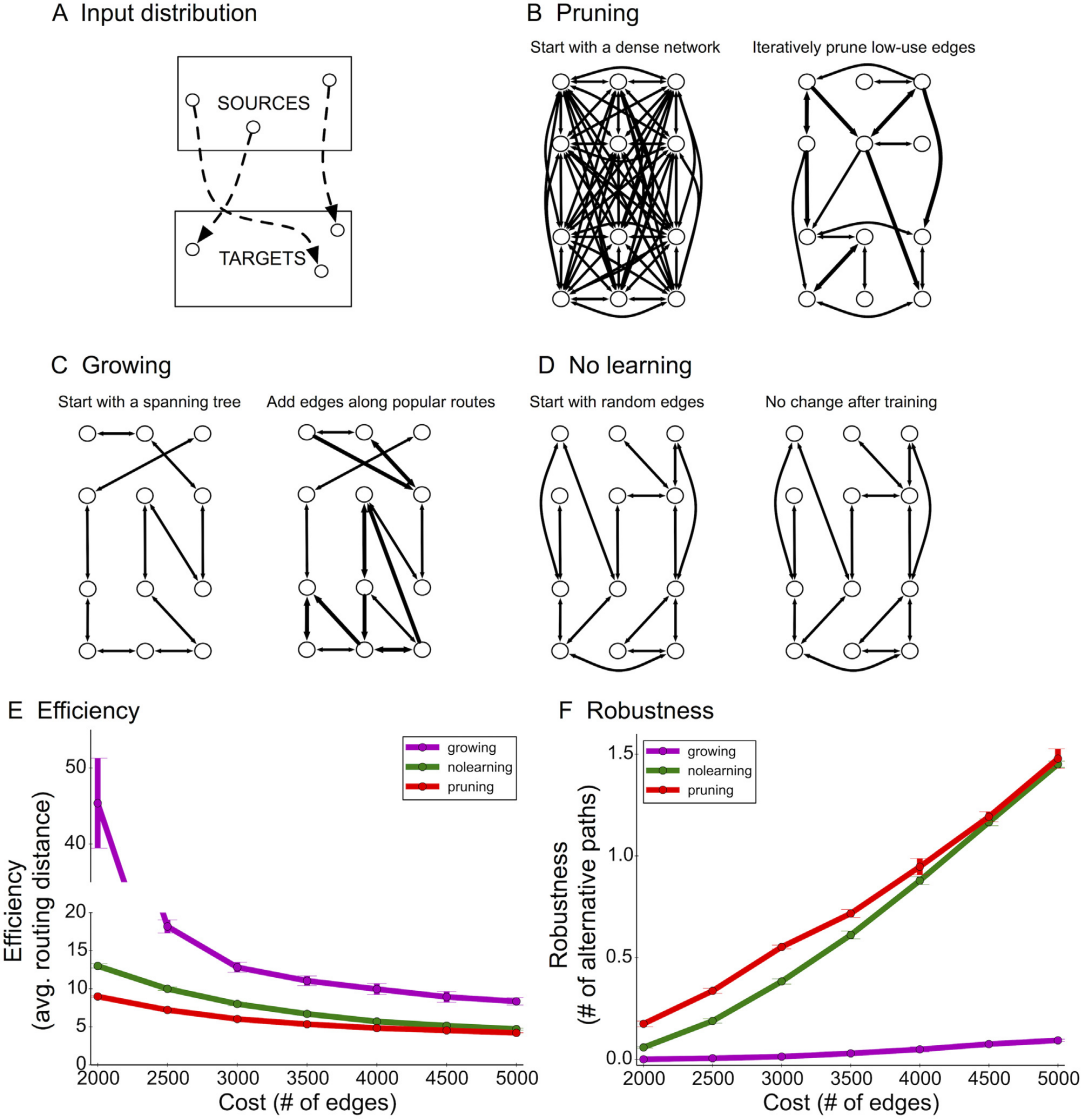
Computational network model and comparison between pruning and growing. (A) Example distribution (2-patch) for source-target pairs. (B) The pruning algorithm starts with an exuberant number of connections. Edges commonly used to route source-target messages are retained, whereas low-use edges are iteratively pruned. (C) The growing algorithm begins with a spanning-tree and adds local shortcut edges along common source-target routes. (D) The no-learning algorithm chooses random edges and does not attempt to learn connections based on the training data. (E+F) Learned networks were evaluated by computing efficiency (E, the average shortest-path distance amongst test pairs) and robustness (F, the average number of short alternative paths between a test source and target). Error bars indicate standard deviation over 3 simulation runs.

Theoretical and practical approaches to engineered network construction typically begin by constructing a basic, backbone network (e.g. a spanning-tree) and then adding connections over time as needed [17]. Such a process is considered cost efficient since it does not introduce new edges unless they are determined to improve routing efficiency or robustness. To quantitatively compare the differences between pruning and growing algorithms, we formulated the following optimization problem: Given *n* nodes and an online stream of source-target pairs of nodes drawn from an *a priori* unknown distribution 𝓓 (Fig 3A), design an efficient and robust network with respect to 𝓓 (Materials and Methods). Efficiency is measured in terms of the average shortest-path routing distance between source-target pairs, and robustness is measured in terms of number of alternative source-target paths (Materials and Methods).

The distribution 𝓓 represents an input-output signaling structure that the network needs to learn during the training (developmental) phase of network construction. This situation occurs in many computational scenarios. For example, wireless and sensor networks often rely on information from the environment, which may be structured but unknown beforehand (e.g. when monitoring river contamination or volcanic activity, some sensors may first detect changes in the environment based on their physical location and then pass this information to other downstream nodes for processing) [24]. Similarly, in peer-to-peer systems on the Internet, some machines preferentially route information to other machines [41], and traffic patterns may be unknown beforehand and only discovered in real-time. In the brain, such a distribution may mimic the directional flow of information across two regions or populations of neurons.

After training, the goal is to output an unweighted, directed graph with a fixed number of edges *B*, representing a limit on available physical or metabolic resources. To evaluate the final network (test phase), additional pairs are drawn from the same distribution 𝓓, and efficiency and robustness of the source-target routes is computed using the test pairs.

Importantly, decisions about edge maintenance, growth, or loss were local and distributed (no central coordinator). The pruning algorithm begins with a dense network and tracks how many times each edge is used along a source-target path. In other words, each edge locally keeps track of how many times it has been used along a source-to-target path. Edges used many times are by definition important (according to 𝓓); edges with low usage values are then iteratively eliminated modeling a “use it or lose it” strategy [42, 43] (Fig 3B). Initially, we assumed elimination occurs at a constant rate, i.e. a constant percentage of existing edges are removed in each interval (Materials and Methods). The growing algorithm first constructs a spanning-tree on *n* nodes and iteratively adds local edges to shortcut common routes [44] (Fig 3C). These algorithms were compared to a fixed global network (no-learning) that selects *B* random directed edges (Fig 3D).

Simulations and analysis of final network structure revealed a marked difference in network efficiency (lower values are better) and robustness (higher values are better) between the pruning, growing, and no-learning algorithms. In sparsely connected networks (average of 2 connections per node), pruning led to a 4.5-fold improvement in efficiency compared to growing and 1.8-fold improvement compared to no-learning (Fig 3E; S8 Fig). In more densely connected networks (average of 10–20 connections per node), pruning still exhibited a significant improvement in efficiency (S7 Fig). The no-learning algorithm does not tailor connectivity to 𝓓 and thus wastes 25% of edges connecting targets back to sources, which does not enhance efficiency under the 2-patch distribution (Fig 3A). Remarkably, pruning-based networks enhanced fault tolerance by more than 20-fold compared to growing-based networks, which were particularly fragile due to strong reliance on the backbone spanning tree (Fig 3F).

### Simulations confirm advantages of decreasing pruning rates

The pruning algorithm employed in the previous simulations used a constant rate of connection loss. Given our experimental results of decreasing pruning rates in neural networks, we asked whether such rates could indeed lead to more efficient and robust networks in our simulated environment. To address this question, the effects of three pruning rates (increasing, decreasing, and constant) on network function were compared (Materials and Methods). Increasing rates start by eliminating few connections and then removing connections more aggressively in later intervals. This is an intuitively appealing strategy since the network can delay edge elimination decisions until more training data is collected. Decreasing rates initially prune aggressively and then taper off over time, which forces earlier decision-making but provides more time for network stabilization.

**Fig 4:**
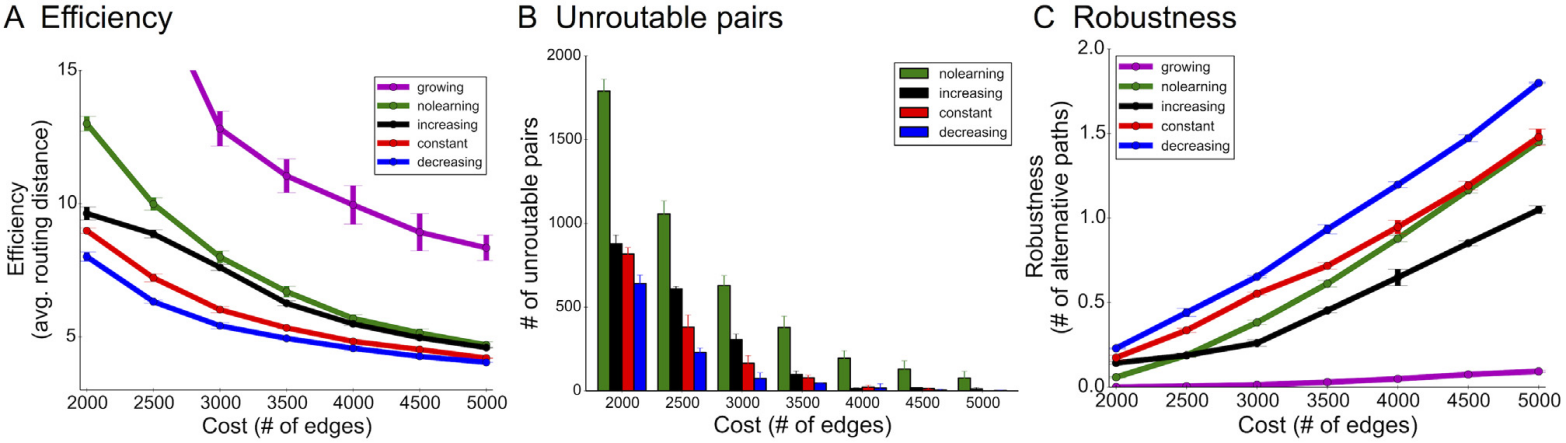
Simulation results for network optimization. (A) Efficiency (lower is better), (B) the number of unroutable pairs (disconnected source-target test pairs), and (C) robustness (higher is better) using the 2-patch distribution. For the growing algorithm, there are no unroutable pairs due to the initial spanning tree construction, which ensures connectivity between every pair to begin with.

Simulations show that the biologically-motivated decreasing rates indeed improve upon the constant rate used previously and created the most efficient and robust networks (Fig 4A–4C). In particular, for the sparsest networks, decreasing rates were 30% more efficient than increasing rates (20% more efficient than constant rates) and exhibited similar gains in fault tolerance. This was particularly surprising because efficiency and robustness are often optimized using competing topological structures: e.g. while alternative paths enable fault tolerance, they do not necessarily enhance efficiency. Further, fewer source-target pairs were unroutable (disconnected from each other) using decreasing rates than any other rate (Fig 4B), which means that these networks were overall better adapted to the activity patterns defined by the distribution 𝓓. Performance of pruning algorithms was also qualitatively similar when starting with sparser initial topologies, as opposed to cliques (S9 Fig).

Interestingly, decreasing rates also consume the least energy compared to the other rates in terms of total number of edges maintained during the developmental period (S10 Fig), which further supports their practical usage.

### An alternative biologically-inspired model for building networks

Neurons likely cannot route signals via shortest paths in networks. To explore a more biologically plausible, yet still abstract, process for network construction, we developed a network-flow-based model that performs a breadth-first search from the source node, which requires no global shortest path computation (Materials and Methods).

**Fig 5:**
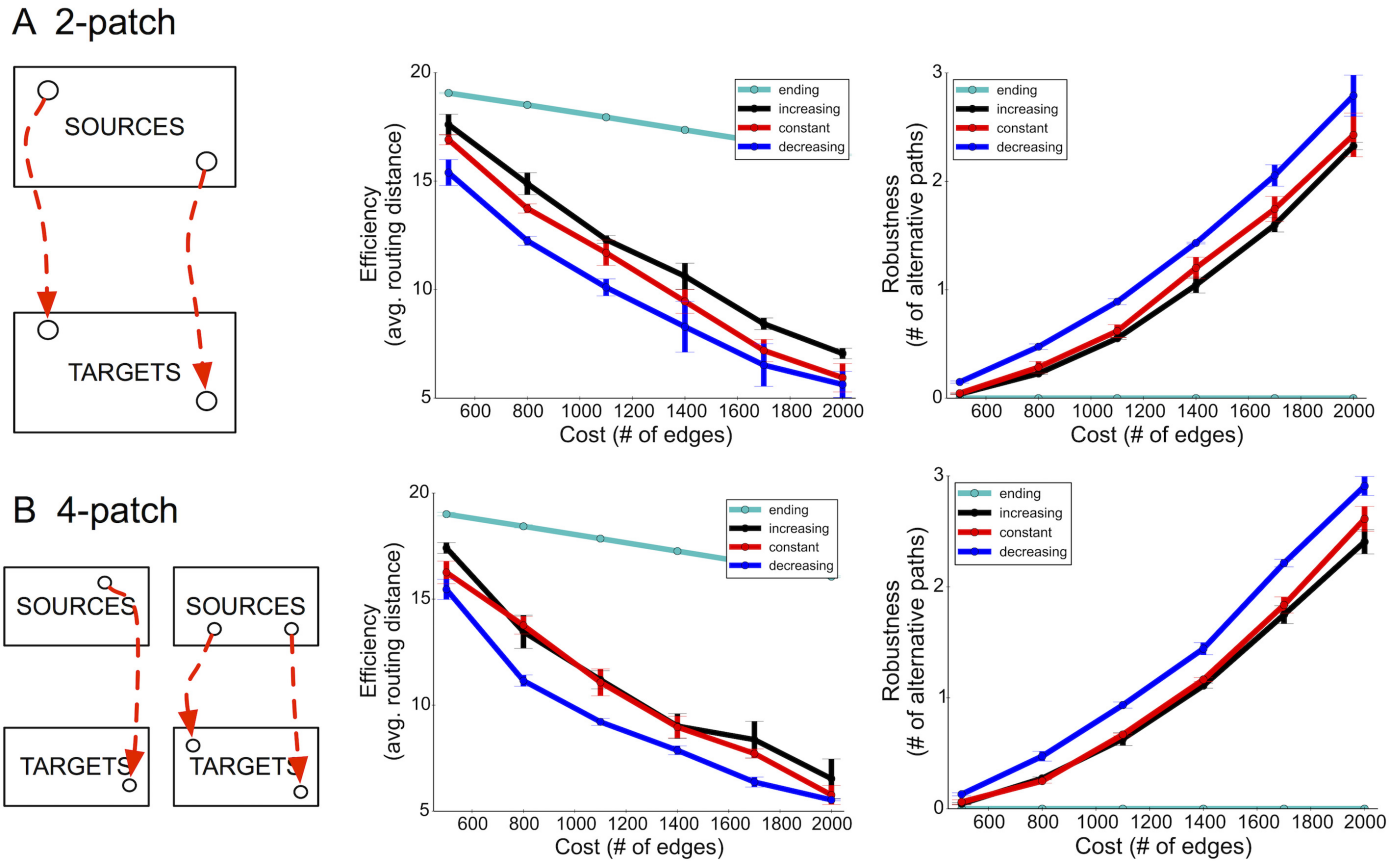
Similar advantages of decreasing pruning rates are observed when using a network-flow-based model of network activity. A) 2-patch input distribution and B) 4-patch input distribution.

Using this model, we see the identical ordering of performance amongst the three rates, with decreasing rates leading to the most efficient and robust networks, followed by constant and then increasing (Fig 5). While our original goal was not to model the full complexity of neural circuits (e.g. using leaky integrate-and-fire units, multiple cell types, etc.), this analysis shows the generality of our biological findings and relevance of pruning rates on network construction.

### Comparing algorithms using additional source-target distributions

**Fig 6:**
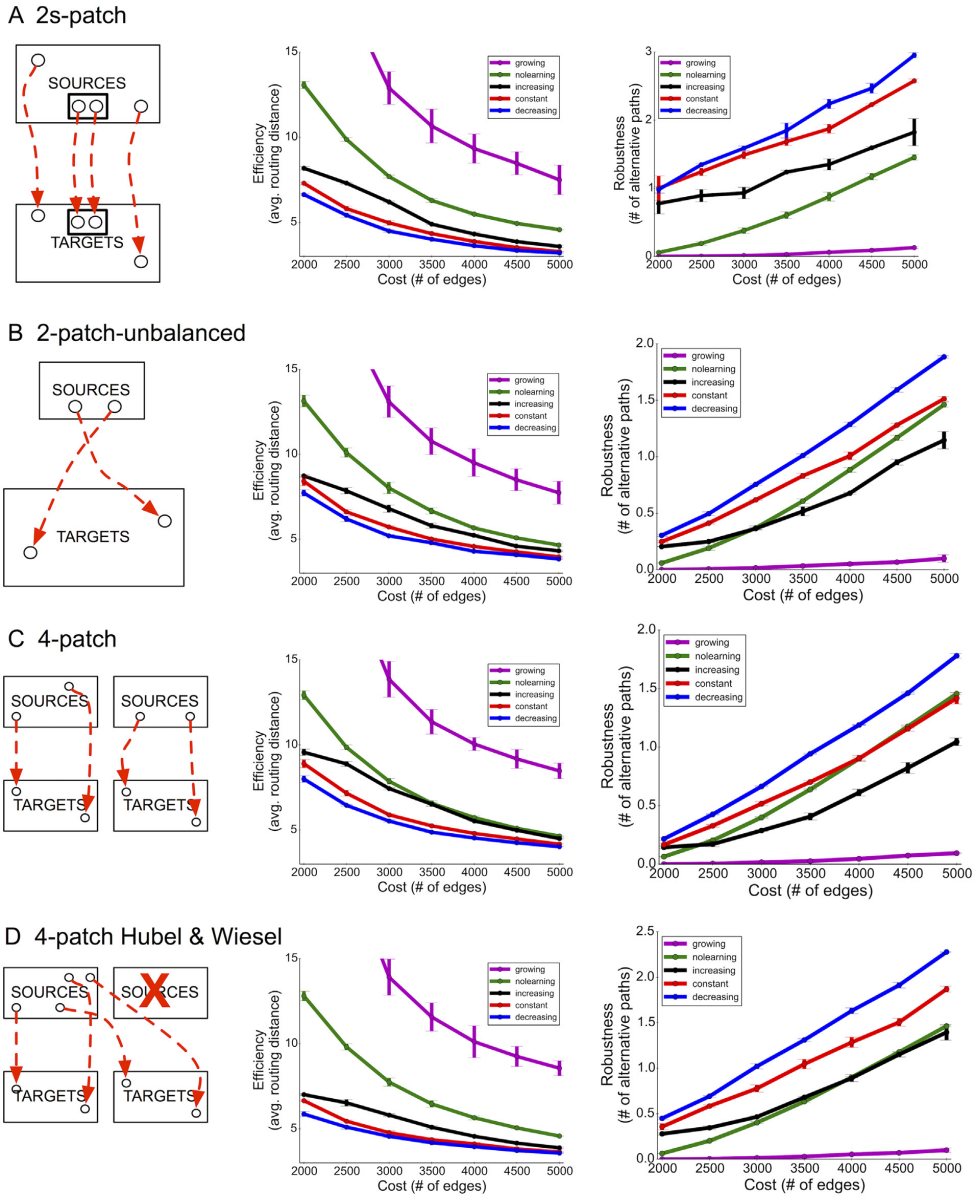
Additional source-target distributions. Decreasing rates are more efficient and robust than all other rates and algorithms for all four distributions: A) 2s-patch. B) 2-patch-unbalanced. C) 4-patch. D) 4-patch Hubel & Wiesel distribution, where during development one input source is lost entirely.

The previous results compared each network construction algorithm using the 2-patch distribution (Fig 3A). This distribution is unidirectional with equal probability of sampling any node within the source and target sets, respectively. Next, we compared each network design algorithm using four additional input distributions. For the 2s-patch distribution (Fig 6A), with probability *x*, a random source and target pair is drawn, but with probability 1−*x*, a random pair is drawn from amongst a smaller more active set of sources and targets. This distribution models recent evidence suggesting highly active subnetworks in the cortex with potentially specialized sources and targets [45, 46]. We set *x* = 0.5 and the size of the selective sets to be 10% each. For the 2-patch-unbalanced distribution (Fig 6B), there are three times as many targets as sources, inspired by the fact that different layers have different numbers of neurons [47]. For the 4-patch distribution (Fig 6C), there are two disjoint sets of sources and targets, each putatively representing input-output activity from adjacent columns or layers. For the 4-patch Hubel-Wiesel distribution (Fig 6D), the second set of sources are shut-off and never drawn from and their corresponding targets are recruited by the first set of sources, mimicking monocular deprivation [16].

Overall, decreasing rates produced the most efficient and robust networks across all distributions. which further supports the generality of our model and experimental observations.

### Analysis of network motifs

To test if our model can replicate statistics of non-random circuits, we detected network motifs within the final network generated using decreasing-rate pruning. We counted all possible 3-node motifs and compared these counts to those expected in a random network [48]. Interestingly, when using the 2-patch distribution, where sources and targets are drawn uniformly from the two sets, we found no over-represented motifs. However, when we considered the 2s-patch distribution (where a subset of sources and targets are selectively more active than the others, as one might expect in real cortical circuits [45, 46, 49, 50]), we found feed-forward motifs to be statistically over-represented when compared to random networks (*P* < 0.01, Z-score = 2.82). This motif has been widely observed in many biological and computational networks and is known for its role in signal propagation and noise control [48].

### Theoretical basis of optimal pruning rates

Given a small, initial sampling of the training source-target pairs, it is relatively easy to determine many connections that will likely not be important. Decreasing rates eliminate these connections quickly, and then provide longer time for the network to fine-tune itself and accommodate indirect pathways while eliminating fewer connections. On the other hand, increasing rates can gather more information early, but then are forced to drastically alter network topology towards the final pruning intervals, which can sever pathways and fragment the network. Interestingly, if the network construction process were guided by a centralized coordinator, then pruning only in the last interval would clearly be a superior strategy because the longer the coordinator waits, the more data is available to determine which edges are most important to inform the centralized design process. However, the distributed nature of the optimization problem forces a different strategy. Indeed, we found more network fragmentation (unroutable pairs) between sources and targets using increasing rates versus decreasing (Fig 4B).

To capture these intuitive notions more formally, we theoretically analyzed the effect of pruning rates on network efficiency. Analysis was simplified in the following way: (1) we only considered efficiency (routing distance) as the optimization target [51]; (2) we assumed the 2-patch routing distribution used for simulation (Fig 3A); and (3) we approximated the topology of the final network using three-parameter Erdős-Rényi random graphs. In these graphs, directed edges between sources 𝓢 → 𝓢 or targets 𝓣 → 𝓣 exist independently with probability *p*, edges from 𝓢 → 𝓣 exist with probability *q*, and edges 𝓣 → 𝓢 existed with probability *z* (S1 Text, S11A Fig; *z* = 0 in optimal sparse networks).

We derived a recurrence to predict the final *p*/*q* ratio given a pruning rate and analytically related the final *p*/*q* ratio to efficiency, the expected path length between source-target pairs (S1 Text, S11B and S11C Fig). Decreasing rates led to networks with near-optimal *p*/*q* ratios, resulting in the best efficiency compared to other rates. Increasing rates yield larger values of *q* (direct source-target edges) because these edges initially represent the shortest routing path for source-target pairs observed during training when the network is very dense. However, these exact pairs are unlikely to be seen again during testing, which leads to over-fitted networks.

From both simulations and theoretical analysis, we found that the regime where decreasing rates are better than increasing rates lies mostly in sparse networks; i.e. where there are 𝓞(*kn*) edges, where *k* is a small constant. For example, with *n* = 1000 nodes, we find *k* in the range of 2–6 to show the most significant differences between rates. This level of sparsity is in line with many real-world geometric networks [52].

### Real-world application to improve airline routing using pruning algorithms

**Fig 7:**
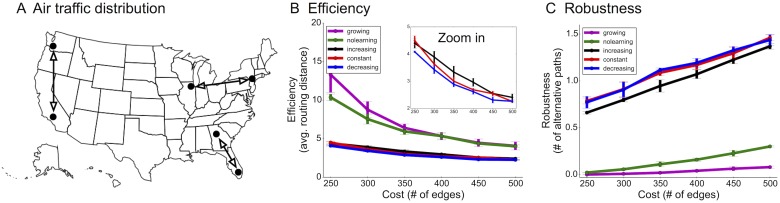
Improving airline efficiency and robustness using pruning algorithms. (A) Actual data of travel frequency amongst 122 popular cities from the 3rd quarter of 2013 was used to define a source-target distribution. (B-C) Efficiency (travel time in terms of number of hops) and robustness (number of alternative routes with the same number of hops) comparison using different algorithms. Decreasing-rate pruning produced more efficient networks with similar robustness.

To demonstrate the utility of decreasing-rate pruning on real-world data, we used it to construct airline routing networks using real traffic data denoting the frequency of passenger travel between US cities. Here, nodes are cities and directed edges imply a direct flight from one city to another (Fig 7A). Due to budgetary constraints, only a subset of routes can be offered based on traffic demands from passengers. We collected data from the Department of Transportation detailing how many passengers flew between the top 1000 source and target city pairs in the United States (e.g. San Francisco to Los Angeles) during the 3rd quarter of 2013 [53]. These frequencies were converted into a distribution (𝓓) denoting the probability of travel between two cities. For this data, a source can also be a target and vice-versa. There were 122 nodes (cities) in the graph. Training and evaluation was done as before.

Decreasing-rate pruning once again outperformed constant and increasing rates, enhancing efficiency by 5–10% with similar robustness when using the same number of edges (Fig 7B and 7C). In other words, these networks can reduce travel time for passengers—especially when travel to some cities is shut down by emergencies—and can reduce overall load of air traffic control systems. While in practice airline routing networks can be designed in a centralized and offline manner, we used this example to show in principle how our technique could work, using real data.

## Discussion

Motivated by new experimental data, we showed that decreasing pruning rates lead to more efficient and robust routing networks compared to other pruning rate strategies and growth-based methods, when learning is distributed and online. While pruning is initially resource-intensive, early hyper-connectivity facilitates rapid convergence to the most important subset of connections. Our experimental and theoretical results may appear counter-intuitive since decreasing rates eliminate more connections early and thus cannot utilize information received later, compared to increasing rates. However, similar to many large-scale engineered systems, the brain is built distributedly, with many concurrent processes that do not have access to a single global planner [54]. Increasing rates prune aggressively at the end, and such last-minute drastic changes in topology leave the network fragmented. Decreasing rates provide the best of both worlds in this regard. They retain extensively used connections and provide more time for the network to fine-tune pathways by making only relatively minor topological modifications in later pruning intervals. Moreover, decreasing rates require the least overall energy to implement because most edges are pruned early in development. This confers an additional practical advantage to their usage. Our results applied to networks designed using both a shortest-path model and a flow-based model.

Simultaneously enhancing both efficiency and robustness, a result achieved by decreasing rates, is not a trivial task. A network in which each node is only connected to a single super-hub can be used to route every source-target pair using at most 2 hops; however, if the primary hub fails, the graph will be entirely disconnected, leading to a fragile network. On the other hand, random networks will have many paths between two nodes, but these paths are not efficient for specific source-target distributions. The fact that decreasing rates outperformed the other rates for both measures attests to its overall power. Given the importance of dynamic construction of distributed networks, for example in wireless computing [55], decreasing-rate pruning may be a viable alternative to current network design methods. These results may be further improved by optimizing the actual rate of decrease in which we prune edges. Further, a more rigorous analysis of the regimes where decreasing rates outperform other rates, including their affect on network robustness, is left for future work.

Prior work studying synapse elimination have primarily focused on the molecular mechanisms controlling this process, including the genes, proteins, and signaling pathways involved [1, 2], and the role of microglia [4]. Quantitative measurements of synaptic density over development have been made in several species, including human (frontal cortex [6], prefrontal cortex [56], visual cortex [57], striate cortex [58]) and mouse (DLGN [59, 60], neuro-muscular junction [61], barrel cortex [9, 33]), amongst others [7, 8]. However, unlike our study that focused on determining pruning rates, the primary goal of these studies was to demonstrate that pruning exists in these areas and to identify the time-period over which it occurs. In some of these studies, decreasing pruning rates can be inferred [56, 58], which further strengthens our findings. However, given their focus as mentioned above, no attempt is made in these prior studies to determine the statistical significance of the observed decreasing rate, and these rates were not linked to network-level information processing (routing), which is our primary contribution. Prior computational modeling of synaptic pruning has used Hopfield networks as an optimization model [5]; while this work also does not analyze pruning rates, our results may shed light on the robustness of memory recall and storage under such a model. Finally, Goyal et al. [62] used expression levels of known synaptic markers to study synapse elimination in human; such expression patterns can potentially also be used to model co-occurring rates of synapse growth and energy consumption (e.g. ATP) during development. There may also be additional pruning parameters important to extract and analyze, such as pruning differences amongst different cell types, the addition of afferents from other brain areas at delayed time-points, and the involvement of glia in synaptic pruning.

Our experimental analysis of pruning rates in the neocortex shows that rates are decreasing over time. This finding has important biological implications for how networks mature during development or reorganize during learning. Given similar levels of activity over the network construction period, these results suggest that the threshold for activation of signaling pathways that initiate synaptic weakening or loss should increase over time. Previous experimental data provides some support to this view, indicating that nascent connections are particularly vulnerable to synaptic depression [63] or elimination [64]. Decreasing pruning rates are also consistent with the developmental time-course of myelination, which shows sharp sigmoidal growth soon after pruning begins [65, 66]. By pruning aggressively early, myelin is not unduly wasted on axons that may ultimately be lost. Clinically, many neurological disorders show abnormal pruning levels during critical development periods—either too many synapses (Fragile X syndrome [67–69]) or too few synapses (Rett syndrome [70–72])—and these phenotypes may also affect network function. While our experimental analysis allowed us to coarsely quantify pruning rates, further challenges remain in longitudinal analysis of synaptic changes within a single animal and automatic synapse detection from large volumes of tissue. Both advances can enable temporally-finer analyses, which can be used to establish more precise pruning rates. Further, any continuous pruning rate that eventually stabilizes will have a time bin over which the rate decreases; our data showed that this decrease persists over multiple days, though finer analyses may be warranted to uncover more precise elimination rates.

Our main goal in this paper was to explore whether a pruning process that mimics how neural networks are formed can be used to construct efficient and robust computational communication networks. To this end, our model abstracted away many other information processing goals of neural networks, including synchronization and transformation of input signals. In addition, we do not model many properties of neural circuits, including connection weights, coincident activation of multiple neurons, spike-timing dependent plasticity, and the presence of inhibitory transmission. Our intention in this study was to highlight the potential importance of pruning rates on global circuit function and to show how this unusual strategy can be applied in various computing applications. Further study will be required to experimentally perturb pruning rates *in vivo* to understand how they affect neural function and behavior.

Our approach of abstracting broad-scale, algorithmic principles from neural networks is likely to provide further insights into the construction of engineered networks and further exemplifies how bi-directional studies can benefit both biology and computer science [11, 73–75].

## Materials and Methods

### Ethics statement

All experiments were carried out in accordance with NIH Guidelines for animal care and use, and were approved by Carnegie Mellon’s institutional animal care and use committee (IACUC protocol AS13-37).

### Electron microscopy imaging and image processing

To experimentally quantify the rate of pruning, we focused on layer 4 of the somatosensory cortex. We extracted, fixed, and sectioned 50*μm*-thick tissue from wildtype C57bl6 (Harlan) mice at different ages. A mitochondrial stain (cytochrome oxidase) was used to visualize the barrelfield, and the D1 barrel was extracted using a dissecting light microscope.

To enable unbiased and high-throughput classification of synapses, we leveraged a staining technique that uses ethanolic phosphotungstic acid (EPTA) to pronounce electron opacity at synaptic sites by targeting proteins in contact zones [38, 39]. This technique typically leaves non-synaptic structures (e.g. plasma membranes, neurotubules, and vesicles) less stained, though considerable variation can exist across samples due to differences in histological chemistry, microscope lighting, etc. Tissue was prepared for electron microscopy (EM) imaging using the same procedure previously described [26]. Both excitatory and inhibitory synapses are stained by this technique [26, 38, 39].

We previously developed a machine learning method that uses support vector machines (SVM) to detect synapses in EPTA-EM images using texture- and shape-based features [26]. The SVM model was trained on data collected in this paper from all 16 developmental time-points. This compromised 3,708 positive examples (synapses) and 39,163 negative examples (non-synapses) across all ages studied. Overall, the classifier was highly accurate and achieved a precision of 90.4% with a recall of 50.0% under 10-fold cross-validation. To ensure that synapse densities were comparable across samples (animals), especially those with variable staining quality, we manually classified synapses in roughly 20 images per sample, applied the classifier (which was built on training data from all the other samples) to these images, and then selected the classification threshold that resulted in 50% recall with 80+% precision (S1 Text, S1 Fig). Recall is defined as: TP / (TP + FN), i.e. the percentage of true synapses correctly predicted by the classifier. Precision is defined as: TP / (TP + FP), i.e. the percentage of predicted synapses that are truly synapses. This means that within each sample, we detected roughly half the synapses, and if the classifier identified a synapse, it was indeed a synapse at least 80% of the time. If precision was < 80% at 50% recall, the sample was removed from the analysis. Table 1 shows average precision and recall values for samples in each time-point. Although we carefully provided our classifier example synapses with a wide variety of structures, shapes, and sizes, there may still be some bias towards classifying certain types of synapses over others. Full details of the imaging method and synapse classification pipeline, including their novelty compared to analysis of conventional electron microscopy images, was previously discussed [26].

A potential method to improve accuracy is to classify synapses in 3D volumes rather than 2D images. Due to challenges related to imaging, alignment, segmentation, and reconstruction across serial sections, such 3D analysis is currently difficult to fully automate [76, 77], which makes it difficult to reason statistically about fine-scale pruning rates. To help control for variability in synapse density in the tissue itself, four regions were sampled from within the barrel (S2 Fig) and counts were averaged. While this approach of sampling multiple regions within the same 2D plane may miss synapses, the same procedure was applied to each animal in each time point, and hence the relative number of synapses per unit area can still be fairly compared to infer a temporal pruning rate.

To perform the statistical analysis of the pruning rates, we binned the data into 12 bins: P14 only, P17 only, P19 only, P21 and P22, P23 and P24, P26 only, P28 only, P30 only, P32 and P33, P34 and P36, P38 only, P40 only. By removing one sample or time-point at a time from the dataset and re-computing the pruning rate using the remaining dataset (known as leave-one-out cross-validation), we statistically determined whether a single sample or time-point was responsible for the observed pruning rate.

### A theoretical framework for distributed network design

We developed a computational model for designing and evaluating distributed routing networks. The problem is as follows:

**Problem:** *Given a set V of n nodes and an online stream of source-target pairs ${\left\{(s_{i},t_{i})\right\}}_{i=1}^{p}$, where s_i_ , t_i_ ∈ V are drawn from some distribution 𝓓, return a graph G with at most B edges that is “efficient” and “robust” with respect to 𝓓.*

The source-target pairs are drawn from an *a priori* unknown distribution 𝓓. This distribution captures some structure in activity (input-output signals) that the network needs to learn during the “training” phase in which the network is constructed. For example, half the nodes can be sources and the other half are targets (the 2-patch distribution; Fig 3A), though the identity of which node belongs to which class is not known a-priori. The sources and targets are individual nodes in the network. The source-target pairs are drawn *online*, which means they are provided one at a time to the network and thus cannot be processed in bulk, mimicking real-time information processing constraints in many types of networks. The pairs are drawn randomly and hence the same pair may appear multiple times in the training or testing sets.

After *p* source-target pairs are seen, the goal is to output an unweighted, directed network *G* with some fixed number of edges (defined as the budget *B*). This budget represents the total allowable cost that the system can maintain (i.e. the number of physical or wireless connections).

### Measuring the quality of a network: Efficiency and robustness

The quality of the final network *G* is evaluated according to its efficiency and robustness when processing an additional *p* pairs drawn from the same distribution 𝓓 (the “testing” phase). During testing, the network is fixed and no changes are made to its connectivity. The test and train pairs may overlap (both are drawn from the same distribution), though they are both likely to also have non-overlapping pairs. This emulates the fact that activity patterns observed during development mimic those expected later but are not exactly the same. Hence, the challenge is to design a network that generalizes the training data and does not over-fit.

Efficiency is defined as the average shortest-path routing distance over all test pairs [78]: $\text{efficiency}(G)=\frac{1}{p}\sum_{u,v\in 𝓓}d(u,v),$ where *p* is the number of source-target pairs observed in the test phase, and *d*(*u*, *v*) is the shortest-path distance between source *u* and target *v* in the final network. If there does not exist a path between a pair, then we set its penalty distance to a large constant.

Robustness is a measure of how tolerant the network is to the deletion of nodes. We adopt a standard measure for robustness based on vertex connectivity [79]: for each source-target pair, we compute the number of alternative paths that have up to one additional hop compared to the true shortest path. This is computed implicitly by removing nodes along the shortest path, and then finding the length of the next shortest path, etc. This definition of robustness ensures that if a primary route is attacked or damaged, an alternative route not only exists but is one that is not much worse than the shortest path.

These definitions are broad, well-established from a graph-theoretic perspective, and applicable to many computing scenarios, but they are not meant to capture all the requirements of information processing in the brain.

### Pruning-based algorithms for distributed network design

To test the impact of pruning and pruning rates we use the following algorithm which is particularly suitable for routing applications. The algorithm begins with a fully connected graph (a clique) on *n* nodes. For each source-target pair, the source routes its message to the target via the shortest path in the graph (computed using a distributed routing table [80, 81]). Initially, all shortest paths will be direct source-to-target paths. Each edge keeps track of the number of times it has been used to satisfy a request(i.e. if an edge *u* → *v* lies on the shortest path from source *s*_*i*_ to target *t*_*i*_, then edge *u* → *v* updates its usage value by 1). All edges initially have a usage of 0.

The above method is appropriate for simulating computational networks. In contrast, neurons likely cannot route signals via shortest paths in networks. We thus tested another simulation model which is more biologically plausible, yet still abstract. Rather than routing, this simulation uses a flow-based model that performs a breadth-first search from the source node (counting all paths between the pairs). Such search does not require any global shortest path computation. In this model, the usage of edges along every successful path that reaches the target is upweighted by 1. This model assumes there is feedback to the circuit that “rewards” every edge active along a source-to-target response [82]. To further mimic synapse failure (signal loss) widely present in neural circuits [83], we assumed a constant signal loss probability of 0.65. This means that with probability 0.65, an edge will fail and will not propagate the signal onwards. Similar values of the signal loss probability led to similar results. This flow process repeats for each source and target during training. Edges are pruned iteratively according to different pruning rates (see below).

For the simulations, the pruning period is divided into 10 discrete intervals, each occurring after 10% of the source-target pairs have been processed. After each interval *i*, some *r*_*i*_-percentage of edges are removed (where *r*_*i*_ depends on the pruning rate, see below). In each interval the pruned edges are those with the lowest-usage (ties are broken randomly).

### Pruning rate strategies

We divided the pruning period into 10 discrete intervals, and after each interval, some *r*_*i*_ percentage of existing connections were pruned. We considered four pruning rate strategies: increasing, decreasing, constant, and ending (S3 Fig).

1. **Constant rate:** *r*_1_ = *r*_2_ = … = *r*_10_. Elimination rates are kept constant (i.e. the same percentage of existing connections are removed in each interval).
2. **Increasing rate:** *r*_1_ < *r*_2_ < … < *r*_10_. Elimination begins very slowly and becomes aggressive later.
3. **Decreasing rate:** *r*_1_ > *r*_2_ > … > *r*_10_. Elimination begins aggressively and then decelerates over time.
4. **Ending rate:** *r*_1_ = *r*_2_ = … = *r*_9_ = 0 and $r_{10}=\frac{B}{n(n-1)}$. Elimination only occurs in the final interval and immediately reduces the network from a clique to exactly *B* edges.

See S1 Text for complete details on how these rates are applied. The Ending rate produced highly overfit networks with only direct edges connecting a subset of source-target pairs seen during training. This yielded the worst efficiency and robustness over all rates.

### Additional network design algorithms: growing and no-learning

We also tested a growth-based algorithm for solving the network design problem that adds connections over time starting from a backbone spanning tree (which are commonly used in engineered systems [17]). See S1 Text for details.

The *no-learning* algorithm simply selects *B* random directed edges to form the final network and ignores the training data.

## Supporting Information

S1 Text Supplementary methods and results. (PDF)

S1 Fig Controlling for image quality in EPTA-EM images. A) First, positive (synapses) and negative (non-synapses) examples were manually labeled in 20 images in the new sample *s*. B) Second, the classifier (trained on images from all other samples, excluding *s*) was applied to the labeled data for *s* and the threshold *τ* that yielded a recall of 50% with precision > 80% was selected. C) Third, the classifier was applied to all images in *s* using *τ* as the classifier threshold.
(TIFF)

S2 Fig Electron microscopy imaging within a barrel. To control for variability in synapse density in different areas in the barrel, 4 regions of the barrel were imaged. Tissue was placed on a mesh copper grid. White circles depict electron beam residue after images were taken. Approximately 240 images per animal (60 images x 4 regions) were taken covering a total of 6,000*μm* ^2^ of tissue per animal.
(TIFF)

S3 Fig Four pruning rate strategies. Constant rates (red) prune an equal percentage of existing connections in each pruning interval. Decreasing rates (blue) prune aggressively early-on and then slower later. Increasing rates (black) are the opposite of decreasing rates. Ending rates only prune edges in the final iteration. A) Number of edges remaining after each pruning interval. B) Percentage of edges pruned in each pruning interval. Here, *n* = 1000.
(TIFF)

S4 Fig Synapse density in adult mice (P65). (TIFF)

S5 Fig Pruning rate with 3D-count adjustment. Adjusted pruning rate per volume of tissue plotted using A) the raw data (where each point corresponds to a single animal) and B) the binned data (where each point averages over animals from a 2-day window).
(TIFF)

S6 Fig Pruning with multiple periods of synaptogenesis and pruning. (TIFF)

S7 Fig Comparing pruning and growing for denser networks. (TIFF)

S8 Fig Comparing the efficiency and robustness of two growing algorithm variants. (TIFF)

S9 Fig Comparing efficiency and robustness of pruning algorithms that start with variable initial connectivity. A) Initial density is 60% (i.e. each edge exists independently with probability 0.6. B) Initial density is 80%.
(TIFF)

S10 Fig Cumulative energy consumed by each pruning algorithm. Energy consumption at interval *i* is the cumulative number of edges present in the network in interval *i* and all prior intervals. Here, *n* = 1000 and it is assumed that the network initially starts as a clique.
(TIFF)

S11 Fig Theoretical results for network optimization. (A) Example edge-distribution using decreasing pruning rates and the 2-patch distribution. (B) Prediction of final network *p*/*q* ratio given a pruning rate. Bold bars indicate simulated ratios, and hashed bars indicate analytical predictions. (C) Prediction of source-target efficiency given a *p*/*q* ratio.
(TIFF)
