# Supplementary material for: Decreasing-Rate Pruning Optimizes the Construction of Efficient and Robust Distributed Networks
Source: PLoS Comput Biol. 2015 Jul 28;11(7):e1004347. doi: 10.1371/journal.pcbi.1004347 (PMC4517947; doi:10.1371/journal.pcbi.1004347)
Supplement: S1 Text — (PDF) [file pcbi.1004347.s001.pdf]

# Decreasing-rate Pruning Optimizes the Construction of Efficient and Robust Distributed Networks

Saket Navlakha[1], Alison L. Barth[2,*], Ziv Bar-Joseph[3,*]
**1 Center for Integrative Biology, The Salk Institute for Biological Studies, La Jolla, CA, 92037 USA.**
**2 Department of Biological Sciences, Center for the Neural Basis of Cognition, Carnegie Mellon University, Pittsburgh, PA 15213 USA**
**3 Lane Center for Computational Biology, Machine Learning Department, Carnegie Mellon University, Pittsburgh, PA 15213 USA.**
**∗ E-mail: barth@cmu.edu, zivbj@cs.cmu.edu**

# Supplementary Methods

## Controlling for variable staining and synapse density

We used a machine learning method that uses support vector machines to detect synapses in ethanolic phosphotungstic acid (EPTA) electron microscopy (EM) images using texture- and shape-based features [1]. The classifier was trained using 3,708 positive examples (synapses) and 39,163 negative examples (non-synapses) across all ages studied. Overall, the classifier was highly accurate and achieved a precision of 90.4% with a recall of 50.0% under 10-fold cross-validation. To ensure that synapse densities were comparable across samples (animals), especially those with variable staining quality, we manually classified synapses in roughly 20 images per sample, applied the classifier (which was built on training data from all the other samples) to these images, and then selected the classification threshold that resulted in 50% recall with 80+% precision (Fig. S1). If precision was $<$ 80% at 50% recall, the sample was removed from the analysis.

**Figure S1. Controlling for image quality in EPTA-EM images.** A) First, positive (synapses) and negative (non-synapses) examples were manually labeled in 20 images in the new sample $s$. B) Second, the classifier (trained on images from all other samples, excluding $s$) was applied to the labeled data for $s$ and the threshold $\tau$ that yielded a recall of 50% with precision $>$ 80% was selected. C) Third, the classifier was applied to all images in $s$ using $\tau$ as the classifier threshold.

To further control for actual variability in synapse density in the tissue itself, four regions were sampled from within the barrel (Fig. S2) and counts were averaged. For each region, roughly 60 images were taken (each image is of size $5\mu m$ by $5\mu m$) covering a total surface area of 6,000 $\mu m^2$ per animal. The images were taken from regions in the same 2D plane.

**Figure S2. Electron microscopy imaging within a barrel.** To control for variability in synapse density in different areas in the barrel, 4 regions of the barrel were imaged. Tissue was placed on a mesh copper grid. White circles depict electron beam residue after images were taken. Approximately 240 images per animal (60 images x 4 regions) were taken covering a total of $6,000\mu m^2$ of tissue per animal.

## Pruning rates and schematics

We divided the developmental pruning period into 10 discrete intervals, and after each interval, some $r_i$ percentage of existing connections were pruned. We considered four pruning rate strategies: increasing, decreasing, constant, and ending (Fig. S3).

1. **Constant rate:** $r_1 = r_2 = \ldots = r_{10}$. Elimination rates are kept constant (i.e. the same percentage of existing connections are removed in each interval).

2. **Increasing rate:** $r_1 < r_2 < \ldots < r_{10}$. Elimination begins very slowly and becomes aggressive later. This strategy imposes longer-term energetic constraints on the network and results in large topological changes towards the end of the pruning period; however, it also provides more time to learn about which connections may be important.

3. **Decreasing rate:** $r_1 > r_2 > \ldots > r_{10}$. Elimination begins aggressively and then decelerates over time. Because many connections are eliminated quickly, this strategy conserves resources overall and allows greater time for stabilization and reinforcement of important connections while making relatively minor topological modifications later in development. However, it also forces convergence to a representative network relatively quickly.

4. **Ending rate:** $r_1 = r_2 = \ldots = r_9 = 0$ and $r_{10} = \frac{B}{n(n-1)}$. Elimination only occurs in the final interval and immediately reduces the network from a clique to exactly $B$ edges.

To set the $r_i$ values for each rate, we do the following: Let $E_0$ be the number of initial edges; then after the first iteration $E_0 \times (1 - r_1)$ edges remain. After the second iteration, $E_0 \times (1 - r_1)(1 - r_2)$ edges remain, etc. For the constant rate, all $r_i$ values are the same and hence can be solved for exactly: $E_0(1 - r_c)^I = B$, where there are $I = 10$ pruning intervals and $B$ is the number of final edges.

For increasing rates, we anchor around the constant rate by removing a lower percentage of edges earlier on and a higher percentage later (Fig. S3). In the first iteration, 10% of edges are removed, and in the last iteration, the corresponding percentage of edges are removed that would equate to two pruning iterations at the constant rate. In other words, by setting $r_1 = 0.1$, we can then solve for $r_{10}$ by: $E(1 - r_c)(1 - r_c) = E(1 - 0.1)(1 - r_{10})$, and so $r_{10} = 1 - \frac{(1-r_c)^2}{0.9}$. In the second iteration, 15% of the edges are removed, and the second-to-last iteration ($r_9$) is similarly adjusted, etc. Overall, we set $r_1 = 0.10, r_2 = 0.15, r_3 = 0.20, r_4 = 0.25, r_5 = r_c$ and then solve for $r_{10}, r_9, r_8, r_7, r_6 = r_c$, respectively. For decreasing rates, the sequence of values used for the increasing rate was reversed. While we naturally observed quantitative differences in efficiency and robustness depending on the exact rate values used for the increasing and decreasing rates, qualitative results remained the same.

While a percentage of edges are removed in each iteration, this can also be implemented in a fully distributed manner by having edges with usage values below a certain threshold remove themselves with an appropriate probability (which will change between iterations).

All strategies begin with $n = 1000$ nodes and end with exactly $B$ edges (which we vary) after seeing $p = 5000$ source-target training pairs.

**Figure S3. Four pruning rate strategies.** Constant rates (red) prune an equal percentage of existing connections in each pruning interval. Decreasing rates (blue) prune aggressively early-on and then slower later. Increasing rates (black) are the opposite of decreasing rates. Ending rates only prune edges in the final iteration. A) Number of edges remaining after each pruning interval. B) Percentage of edges pruned in each pruning interval. Here, $n = 1000$.

# Supplementary Results

## Pruning in adult mice

To determine whether pruning continued after P40 into adulthood, we imaged 3 additional mice at age P65. Fig. S4 shows that synapse density at P40 had indeed stabilized to levels observed in adulthood.

**Figure S4. Synapse density in adult mice (P65).**

## Potential confounding factors in synapse density estimation

Volumetric differences in the brains of developing mice could be one possible confounding factor when estimating synapse density. Cortical thickness changes substantially from P5–P15 [2,3], but our experiments were performed at later time-points, and hence we do not expect this to significantly affect our results. Age-dependent variability may also arise from differences in pruning rates for excitatory and inhibitory neurons, but the number of inhibitory synapses in the barrel cortex is also believed to stabilize by P15 [4].

Further, to remove some biases in 2D analysis caused by larger synapses, previous works have proposed formulas to adjust counts based on the average size of synaptic profiles observed in the sample [5]. For example, a popular measure of adjustment is based on the Abercrombie correction [6, 7]. This adjustment is computed as follows: $N_V = N_A \times T/(D+T)$, where $N_A$ is the number of synapses per unit area (image), $T$ is the section thickness ($100nm$), $D$ is the mean length of synaptic profiles in the sample, and $N_V$ is the estimated number of synapses per unit volume. We inferred profile lengths directly from the images [1] and applied the adjustment to each sample. Overall, we still observe a decreasing pruning rate (Fig. S5).

**Figure S5. Pruning rate with 3D-count adjustment.** Adjusted pruning rate per volume of tissue plotted using A) the raw data (where each point corresponds to a single animal) and B) the binned data (where each point averages over animals from a 2-day window).

## Potential for multiple decays during developmental pruning

While we assumed a single pruning process, our data indicates that there may be an additional round of synaptogenesis at P33 followed by a second pruning period. To test the rates implied by this possibility, we fit two pruning curves split between P19-P32 and P33-P40 (Fig. S6). The first curve continues to show a decreasing rate of pruning: 24%, 21%, and 12% of synapses are pruned in successive pruning intervals during this period. The second pruning curve shows an initial decreasing rate of pruning (13% to 9%), followed by an increase in the last interval (13%). This data, however, is very difficult to interpret because of the short time interval for the second curve. For such a short duration the differences between decreasing and constant pruning rates are minimal (since the number pruning intervals and pruning events is much smaller than for the longer-duration curve). Further, the new synapses may only undergo minor pruning, and the additional pruning observed may represent continued pruning of the original synapses. Thus, it is difficult to tease these two rates apart from our data.

We also computed $\tau$, the exponential time decay constant, of the interpolated pruning curve from P19 to P32. We found that roughly two-thirds of the total pruning occurred within the first $\tau = 5.8$ days, which further suggests that the majority of elimination is occurring quickly.

**Figure S6. Pruning with multiple periods of synaptogenesis and pruning.**

## Comparing pruning vs. growing at higher cost values

To show that differences in efficiency and robustness between growing and pruning persist for denser final networks, we built networks with 1000 nodes and 10-20,000 edges (i.e. 10-20 connections per node, compared to 2-5 connections per node shown in the main text). Even in this denser regime, we find significant benefit of the pruning algorithm (Fig. S7).

**Figure S7. Comparing pruning and growing for denser networks.**

## Comparing two variants of growing

The *growing* algorithm starts with a random directed spanning tree of the graph computed as follows: initially, all nodes belong to their own isolated component and in each step, two random nodes $u$ and $v$ from two different components are selected, and the edges $u \to v$ and $v \to u$ are added. This process repeats until a single strongly connected component is formed, leading to a graph with $2n - 2$ edges.

The remaining $B - 2n - 2$ edges are added during the training phase. Like the pruning algorithms, sources are routed to their targets via the shortest path in the graph and edge-usage values are maintained and updated locally. In particular, after every $p/(B - 2n - 2)$ training pairs are processed, a new edge is added to the graph as follows: we find the highest-use edge $u \to v$ in the graph and the highest-use neighbor $v'$ of $v$ and add a shortcut edge $u \to v'$. Such a shortcut improves the future efficiency of messages that are routed along this already-popular path. This process produces many feed-forward directed edges by closing triangles, which is a common mechanism of growth in other types of complex networks [8].

We tested also another growing algorithm that starts with the minimum number of directed edges required to ensure connectivity between all pairs of $n = 1000$ nodes. This network contains 999 edges (consisting of a chain with a loop from the last node back to the first node). Similar to the original growing algorithm, we added local edges to the tree along popular routes, as can be implemented in a distributed fashion. We found that the original growing algorithm was much more efficient than the modified version (called *growingalt*) across the entire range of costs (Fig. S8). This gain can be attributed to the fact that the modified growing algorithm initially may need to traverse $n - 1$ edges to route a request in the worst-case. Thus, convergence to more appropriate networks is faster for the original growing algorithm.

**Figure S8. Comparing the efficiency and robustness of two growing algorithm variants.**

## Performance of pruning algorithms that do not start with cliques

How dependent is the performance of our algorithms on the density of the initial graph? To test this, instead of starting with a clique, we started with sparser networks. In particular, instead of each edge existing with probability $p = 1$, we test initial configurations where $p = 0.6$ or $p = 0.8$. In both cases, we see very similar qualitative results and trends across all algorithms (Fig. S9).

**Figure S9. Comparing efficiency and robustness of pruning algorithms that start with variable initial connectivity.** A) Initial density is 60% (i.e. each edge exists independently with probability 0.6. B) Initial density is 80%.

## Analysis of energy consumption by pruning algorithms

Reducing energy consumption is also important in the brain. If energy consumption is measured as the cumulative number of edges (synapses) maintained during the training period (development), then because decreasing rates eliminate many edges early (compared to increasing and constant rates), decreasing rates also consume the least overall energy during learning (Fig. S10).

**Figure S10. Cumulative energy consumed by each pruning algorithm.** Energy consumption at interval $i$ is the cumulative number of edges present in the network in interval $i$ and all prior intervals. Here, $n = 1000$ and it is assumed that the network initially starts as a clique.

## Theoretical comparison of different pruning rates

To theoretically analyze the properties of the optimal routing network and to compare such properties to those obtained by each of the network design strategies we simplified the analysis in the following ways: (1) we only consider efficiency (routing distance) as the optimization target [9]; (2) we assumed the 2-patch routing distribution used for the simulation; and (3) we approximated the topology of the output network using three-parameter Erdős-Rényi random graphs. In these graphs, each edge connecting two source nodes $\mathcal{S}$ or target nodes $\mathcal{T}$ exists independently with probability $p$, each edge from $\mathcal{S} \to \mathcal{T}$ exists with probability $q$, and each edge from $\mathcal{T} \to \mathcal{S}$ exists with probability $z$. First, we show that in the optimal (sparse) network, where there are $\mathcal{O}(kn)$ edges ($k \ll n$; for example, in the range 2–6), $z = 0$.

**Theorem 1.** *In the optimal sparse network, there are no directed edges from $\mathcal{T} \to \mathcal{S}$, i.e. $z = 0$.*

*Proof.* By contradiction, consider a path that uses such an edge. This path will consist of at least 4 nodes: $s_1 \to t_1 \to s_2 \to t_2$. Clearly, this path can be made shorter by replacing the intermediate edge $t_1 \to s_2$ with $s_1 \to s_2$ or equivalently, by replacing $t_1 \to s_2$ with $t_1 \to t_2$. Hence, in the optimal network, there should be no edges of this type.

□

Next, we show that different $p/q$ ratios result in different expected path lengths between random source-target pairs (step 1), and then we show that decreasing pruning rates get closer to the optimal ratio of $p/q$ than either increasing or constant rates (step 2).

### Step 1: source-target connectivity in two-parameter Erdős-Rényi random graphs

Each edge in the network exists independently, thus on average we expect $2pn(n-1)$ total directed edges between nodes in $\mathcal{S}$ and $\mathcal{T}$, respectively and $qn^2$ edges from $\mathcal{S}$ to $\mathcal{T}$.

Consider the shortest-path distance between a fixed source node $s \in \mathcal{S}$ and target node $t \in \mathcal{T}$. Let $F_d$ be the probability that $dist(s,t) > d$, i.e. the shortest-path distance between $s$ and $t$ in $G$ is greater than $d$. This is equivalent to the probability that there is no path of length $\leq d$ from $s$ to $t$. Below, we derive a recurrence relation that allows us to compute $F_d$ for any value of $d$ following Blondel et al. [10].

The relation $dist(s,t) > d$ holds if $t$ is not connected to any node at distance $< d$ from $s$. Because of the different connection probabilities depending on whether the node is in $\mathcal{S}$ and $\mathcal{T}$, we split this probability into two pieces:

$$F_d = \sum_{k=1}^{n-1} P[n_d^S = k](1-q)^k \sum_{k=1}^{n-1} P[n_d^T = k](1-p)^k, \tag{1}$$

where $n_d^S$ is the number of nodes in $\mathcal{S}$ at distance $< d$ from $s$, $n_d^T$ is the same for nodes in $\mathcal{T}$, and $P[n_d = k]$ denotes the probability that $n_d = k$. We approximate each $n_d$ distribution by its expectation so that $n_d$ equals the probability a node is distance $< d$ multiplied by $n$.

$$n_d^S = (1 - F_{d-1}^S)n \tag{2}$$

$$n_d^T = (1 - F_{d-1}^T)n. \tag{3}$$

Putting these together:

$$F_d = (1-q)^{n_d^S}(1-p)^{n_d^T} \tag{4}$$

$$= (1-q)^{(1-F_{d-1}^S)n}(1-p)^{(1-F_{d-1}^T)n}. \tag{5}$$

The terms $F_d^S$ and $F_d^T$ correspond to the $F_d$ probabilities with respect to nodes in $\mathcal{S}$ and $\mathcal{T}$, respectively. To calculate these, consider a random node in $\mathcal{S}$. This node is at distance $> d$ from $s$ if it is not the

**Figure S11. Theoretical results for network optimization.** (A) Example edge-distribution using decreasing pruning rates and the 2-patch distribution. (B) Prediction of final network $p/q$ ratio given a pruning rate. Bold bars indicate simulated ratios, and hashed bars indicate analytical predictions. (C) Prediction of source-target efficiency given a $p/q$ ratio.

source itself, which happens with probability $(1 - 1/n)$, and if it is not connected to any node that is at distance $< d$ from $s$, which happens with probability $(1-p)^{(1-F^S_{d-1})n}$. Similarly, consider a random node in $\mathcal{T}$. This node is at distance $> d$ from $s$ if it is not connected to any node in $\mathcal{S}$ (via an edge across the cut, existing with probability $q$) nor $\mathcal{T}$ (via a lateral edge, existing with probability $p$) that is distance $< d$ from $s$. Thus:

$$F^S_d = (1 - 1/n)(1 - p)^{(1-F^S_{d-1})n} \tag{6}$$

$$F^T_d = (1 - q)^{(1-F^S_{d-1})n}(1 - p)^{(1-F^T_{d-1})n}. \tag{7}$$

The base case for these recurrences are: $F^S_0 = 1 - 1/n$ (i.e. a random node in $\mathcal{S}$ is at distance $> 0$ from $s$ only if it is not $s$ itself) and $F^T_0 = 1$ (i.e. every node in $\mathcal{T}$ is at least one hop from $s$).

The probability that $s$ and $t$ are exactly $d$ hops away is given by $f_d = F_{d-1} - F_d$. From these relations, we can compute the expected distance between $s$ and $t$ as: $\sum_{d=1}^{n} d \times f_d$.

To test the correspondence between theory and practice, we generated random two-parameter Erdős-Rényi graphs with various ratios of $p/q$ such that the expected number of edges is approximately $m = 6000$ with $n = 1000$. For each ratio, we computed the average distance between random s-t pairs and compared these values with those predicted by our theoretical results above and found very close correspondence (Fig. S11C).

### Step 2: predicting $p/q$ ratios for different pruning strategies

In step 1, we established a link between the $p/q$ ratio and the expected source-target connectivity. Next, we derive a recurrence to compute the $p/q$ ratios for any given pruning rate.

Our idea is to predict, in each iteration, how many $\mathcal{S} \to \mathcal{S}$, $\mathcal{T} \to \mathcal{T}$, and $\mathcal{S} \to \mathcal{T}$ edges will be used to route source-target requests. This will allow us to calculate how many edges in each class will be pruned, which will allow us to estimate the values of $p$ and $q$ after each iteration. By repeating this procedure for each pruning iteration, we can estimate the final values of $p$ and $q$.

Say we can calculate the values of $p$ and $q$ in iteration $i$; then from step 1 above we can estimate the expected path length $l_i$ for all source-target requests in the following iteration. In every such s-t path, exactly one $\mathcal{S} \to \mathcal{T}$ edge will be used (because no backwards $\mathcal{T} \to \mathcal{S}$ edges exist) and the remaining $l_i - 1$ edges will be split evenly amongst $\mathcal{S} \to \mathcal{S}$ edges and $\mathcal{T} \to \mathcal{T}$ edges. Initially, $p_0 = q_0 = 1$ (we start with a clique) and the expected path length is 1 and only $\mathcal{S} \to \mathcal{T}$ edges will be updated.

For simplicity, assume that if an edge is used once, it will be safe from being pruned in all subsequent pruning iterations. (Though this assumption can be relaxed, it will likely be the case anyways for sufficiently large $B$.) Then, our goal is to keep track of 0-use edges in each edge class ($\mathcal{S} \to \mathcal{S}$, $\mathcal{T} \to \mathcal{T}$, and $\mathcal{S} \to \mathcal{T}$) and prune edges from this set proportionally.

Let $f_p(0, i)$ be the number of edges with weight $\geq 0$ from $\mathcal{S} \to \mathcal{S}$ (or equivalently, $\mathcal{T} \to \mathcal{T}$) after iteration $i$, and let $f_q(0, i)$ be the same quantity for $\mathcal{S} \to \mathcal{T}$ edges. The total number of 0-use edges in the network in iteration $i$ is: $f(0, i) = 3n^2 - f_p(1, i) - f_q(1, i) - \sum_{k=1}^{i} r'_k$. There are approximately $3n^2$ total edges to start; the second and third terms are the number of edges amongst the $\mathcal{S} \to \mathcal{S}$ and $\mathcal{S} \to \mathcal{T}$ classes that have been used at least once, and the fourth term is the number of edges pruned thus far ($r'_k$ is the number of edges pruned in the $k^{\text{th}}$ interval). Initially, $f_p(0, 0) = f_q(0, 0) = n^2$.

In the $i^{\text{th}}$ iteration:

$$f_q(0,i) = f_q(0,i-1) - r_i\Big(\frac{f_q(0,i-1) - f_q(1,i)}{f(0,i)}\Big). \tag{8}$$

The first term on the RHS is the current number of 0-weight edges from $\mathcal{S} \to \mathcal{T}$. From this, we subtract a proportion $r_i$ of the total number of edges that we want to prune in iteration $i$. This proportion is equal to the new (reduced) number of 0-weight edges from $\mathcal{S} \to \mathcal{T}$ divided by the total number of 0-weight edges in the entire network. A similar equation is used to calculate $f_p(0,i)$.

To calculate $f_q(1,i)$ (the number of edges used at least once from $\mathcal{S} \to \mathcal{T}$) we start with the base case: $f_q(1,0) = p/10$, i.e. prior to the first iteration, exactly $p/10$ direct $\mathcal{S} \to \mathcal{T}$ edges have been used, and none others. The base cases for the $\mathcal{S} \to \mathcal{S}$ edges are: $f_p(1,0) = 0$ (again, all routes are initially direct because we start with a clique) and $f_p(1,1) = (l_i - 1)/2 \times p/10$. Here, $l_i$ is the expected s-t distance derived from step 1 given the current values of $p$ and $q$. In this path, exactly one edge will be used to go from $\mathcal{S} \to \mathcal{T}$ and the remaining edges will be split evenly amongst $\mathcal{S} \to \mathcal{S}$ and $\mathcal{T} \to \mathcal{T}$ edges.

In the $i^{\text{th}}$ iteration:

$$f_q(1,i) = f_q(1,i-1) + hyper(N = f_q(0,i-1), m = f_q(1,i-1), n = p/10) \tag{9}$$

The first term on the RHS is the number of $\geq$ 1-weight edges in the previous iteration. Some of the edges used in the next round may overlap with these edges. The function *hyper*() calculates the expected number of *new* edges that were previously of 0-weight but now have weight $\geq 1$ using the hypergeometric formula.

After iteration 10, the final values of $p$ and $q$ are $f_p(0,10)/n^2$ and $f_q(0,10)/n^2$, respectively. The only variable are the $r_i$ values that change depending on the pruning rate.

Given these recurrence formulas, we can compute the ratio of $p/q$ for the final networks for each of the three pruning strategies (Fig. S11B). We find very close correspondence between the theoretically-derived ratios and the ratios obtained by the simulations. Decreasing rates produce networks with near-optimal $p/q$ ratios, whereas increasing rates tend to have larger values of $q$ due to over-fitting to the training data.

Given a specific value for $n$ (number of nodes) and the final connectivity (total number of edges), our theoretical analysis can estimate which pruning rate leads to the best network efficiency (shortest-path routing distance). By first applying Step 2, we can determine the expected $p/q$ ratio for each rate. Then by applying Step 1, we can determine the expected efficiency of the network given this $p/q$ ratio. This analysis may be useful for several of the applications we outline in the Introduction.

# References

1. Navlakha S, Suhan J, Barth AL, Bar-Joseph Z (2013) A high-throughput framework to detect synapses in electron microscopy images. Bioinformatics 29: 9–17.

2. Agmon A, Yang LT, O'Dowd DK, Jones EG (1993) Organized growth of thalamocortical axons from the deep tier of terminations into layer IV of developing mouse barrel cortex. J Neurosci 13: 5365–5382.

3. Fox K (2008) Barrel Cortex. Cambridge University Press.

4. Micheva KD, Beaulieu C (1996) Quantitative aspects of synaptogenesis in the rat barrel field cortex with special reference to GABA circuitry. J Comp Neurol 373: 340–354.

5. Coggeshall RE, Lekan HA (1996) Methods for determining numbers of cells and synapses: a case for m ore uniform standards of review. J Comp Neurol 364: 6–15.

6. Mayhew TM (1979) Stereological approach to the study of synapse morphometry with particular regard to estimating number in a volume and on a surface. J Neurocytol 8: 121–138.

7. Huttenlocher PR, Dabholkar AS (1997) Regional differences in synaptogenesis in human cerebral cortex. J Comp Neurol 387: 167–178.

8. Leskovec J, Backstrom L, Kumar R, Tomkins A (2008) Microscopic evolution of social networks. In: Proc. 14th ACM SIGKDD Intl. Conf. on Knowledge Discovery and Data Mining (KDD). New York, NY, USA: ACM, KDD '08, pp. 462–470.

9. Royer E, Toh CK (1999) A review of current routing protocols for ad hoc mobile wireless networks. Personal Communications, IEEE 6: 46-55.

10. Blondel VD, Guillaume JL, Hendrickx JM, Jungers RM (2007) Distance distribution in random graphs and application to network exploration. Phys Rev E 76: 066101.
