# Supplementary figures and images for: Decreasing-Rate Pruning Optimizes the Construction of Efficient and Robust Distributed Networks
Source: PLoS Comput Biol. 2015 Jul 28;11(7):e1004347. doi: 10.1371/journal.pcbi.1004347 (PMC4517947; doi:10.1371/journal.pcbi.1004347)

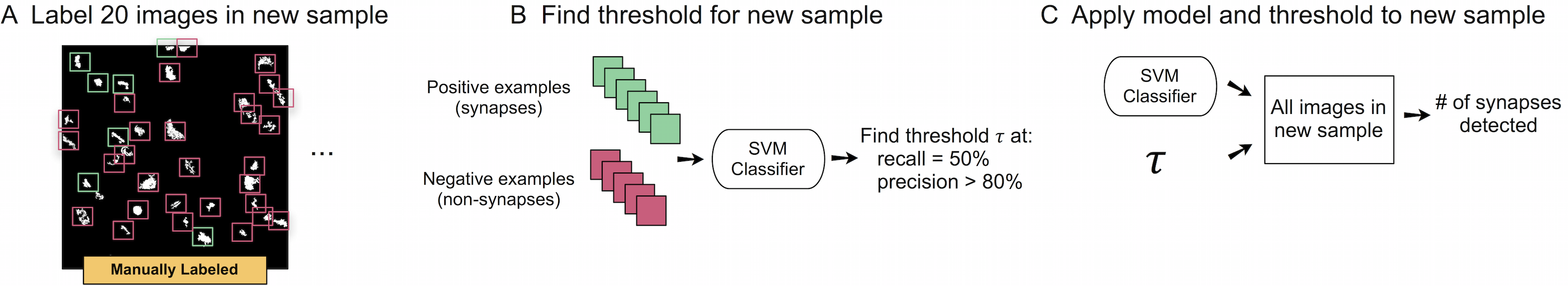

Supplement: S1 Fig — A) First, positive (synapses) and negative (non-synapses) examples were manually labeled in 20 images in the new sample s. B) Second, the classifier (trained on images from all other samples, excluding s) was applied to the labeled data for s and the threshold τ that yielded a recall of 50% with precision > 80% was selected. C) Third, the classifier was applied to all images in s using τ as the classifier threshold. (TIFF) [file pcbi.1004347.s002.tiff]

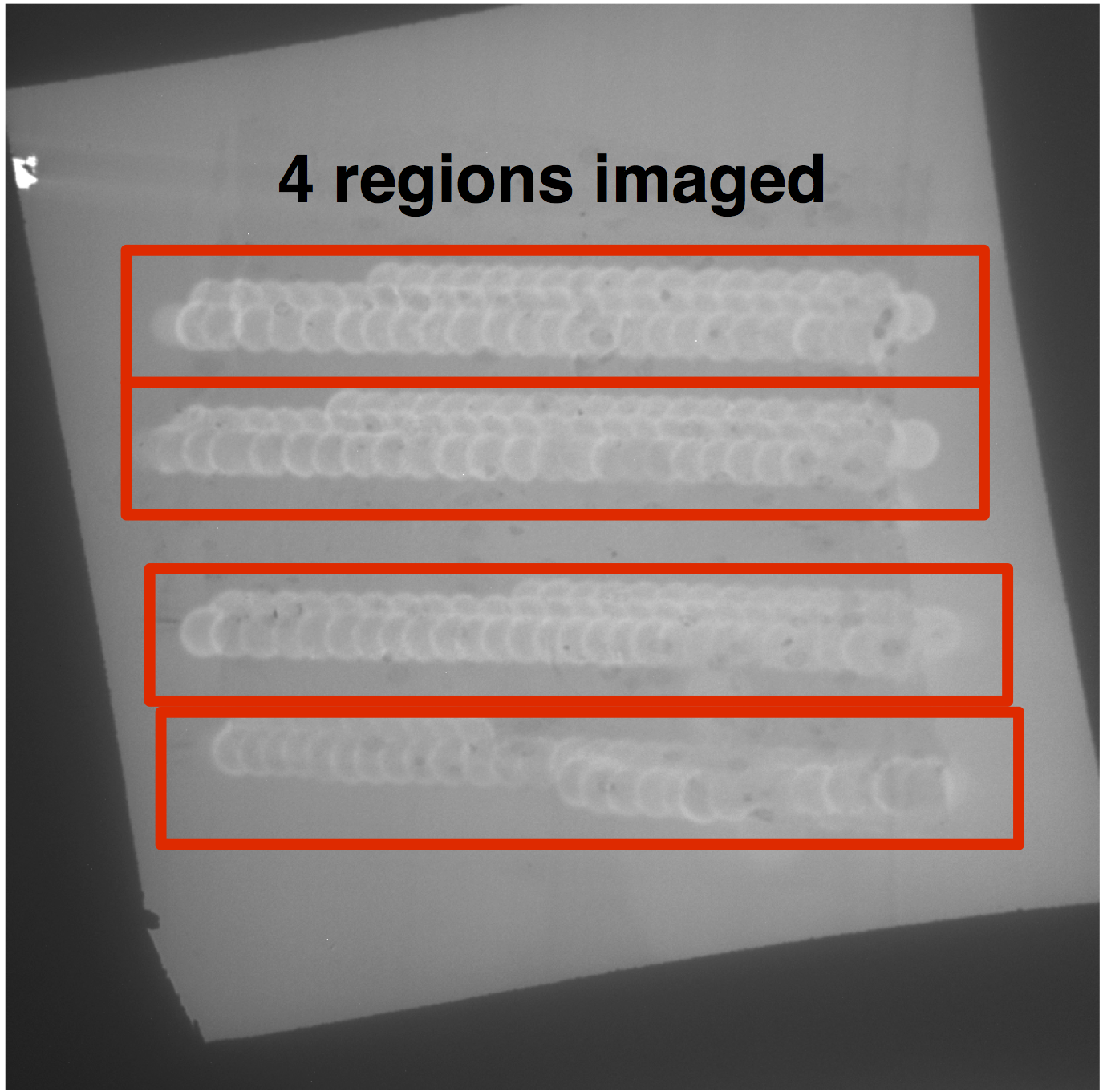

Supplement: S2 Fig — To control for variability in synapse density in different areas in the barrel, 4 regions of the barrel were imaged. Tissue was placed on a mesh copper grid. White circles depict electron beam residue after images were taken. Approximately 240 images per animal (60 images x 4 regions) were taken covering a total of 6,000μm 2 of tissue per animal. (TIFF) [file pcbi.1004347.s003.tiff]

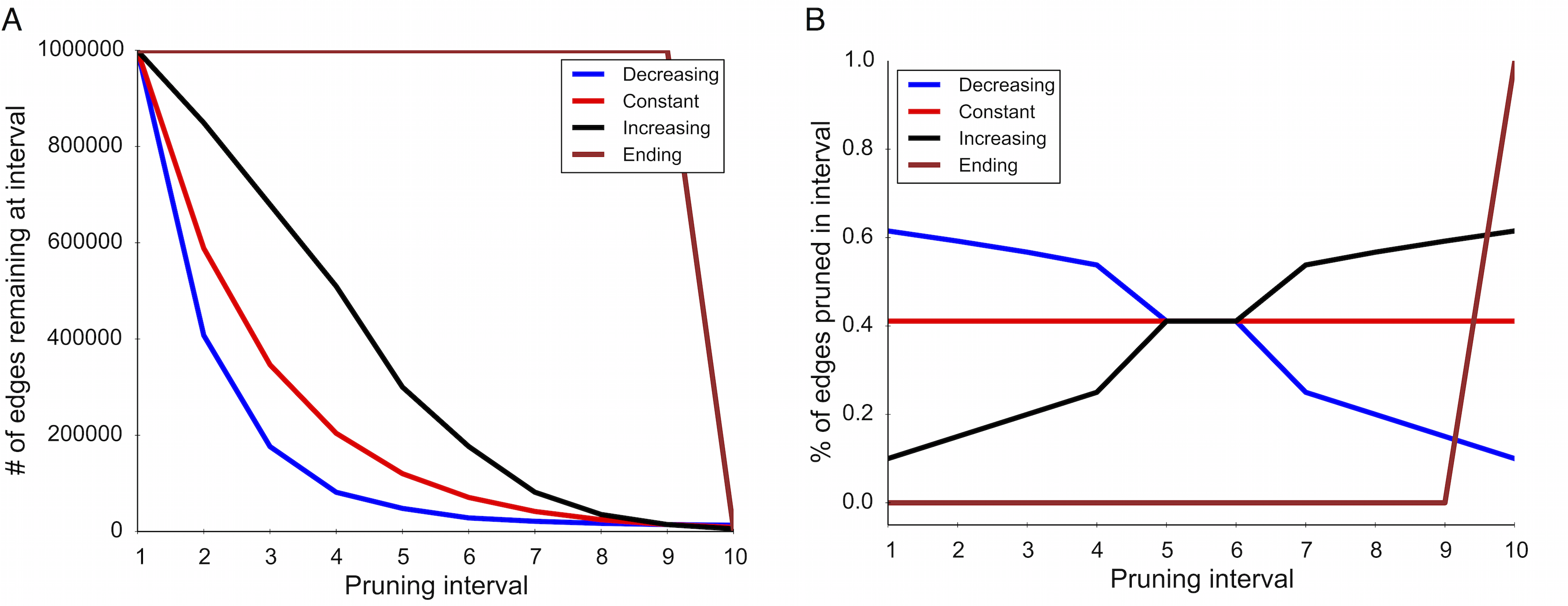

Supplement: S3 Fig — Constant rates (red) prune an equal percentage of existing connections in each pruning interval. Decreasing rates (blue) prune aggressively early-on and then slower later. Increasing rates (black) are the opposite of decreasing rates. Ending rates only prune edges in the final iteration. A) Number of edges remaining after each pruning interval. B) Percentage of edges pruned in each pruning interval. Here, n = 1000. (TIFF) [file pcbi.1004347.s004.tiff]

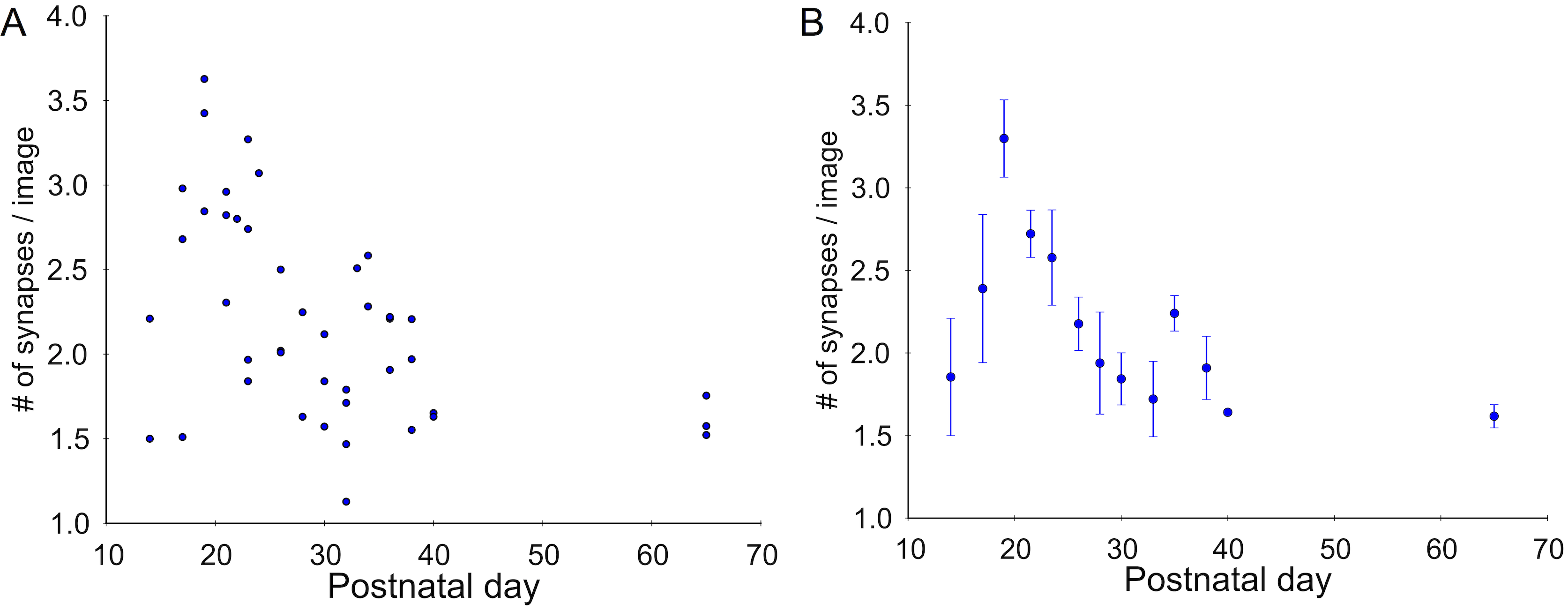

Supplement: S4 Fig — (TIFF) [file pcbi.1004347.s005.tiff]

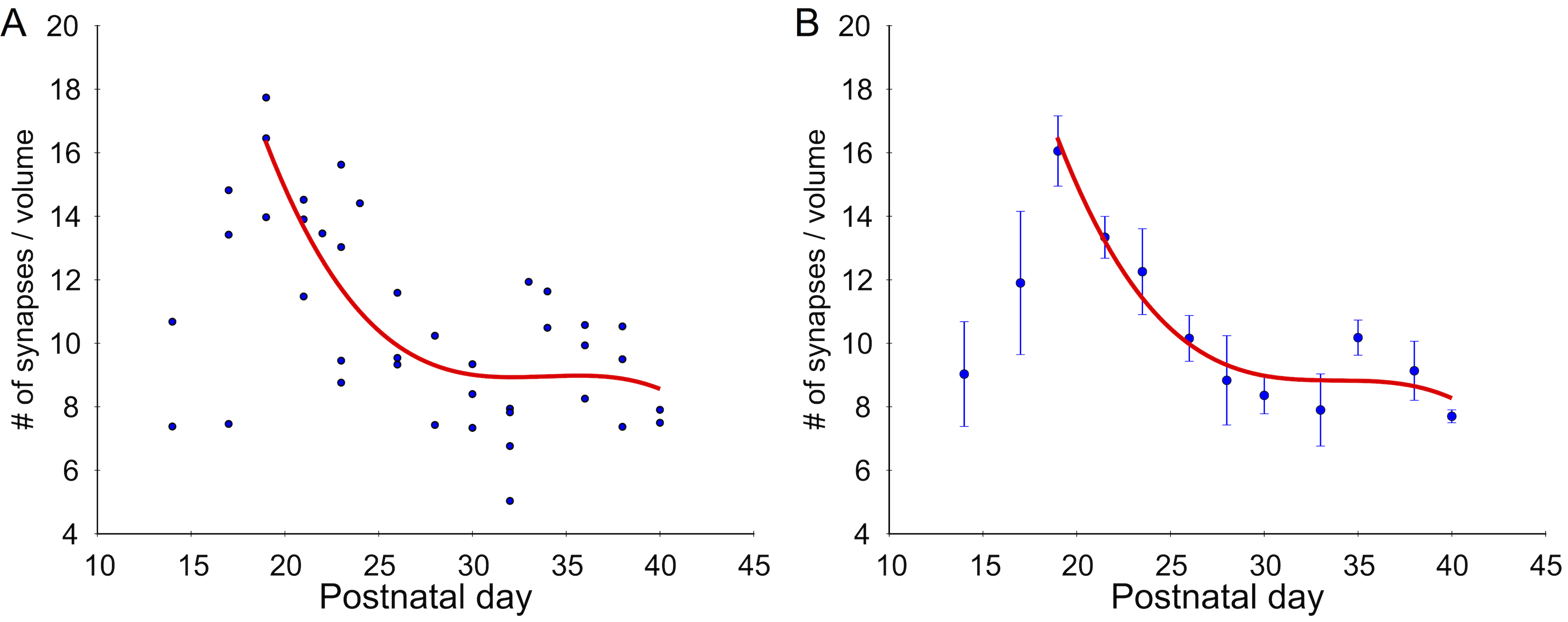

Supplement: S5 Fig — Adjusted pruning rate per volume of tissue plotted using A) the raw data (where each point corresponds to a single animal) and B) the binned data (where each point averages over animals from a 2-day window). (TIFF) [file pcbi.1004347.s006.tiff]

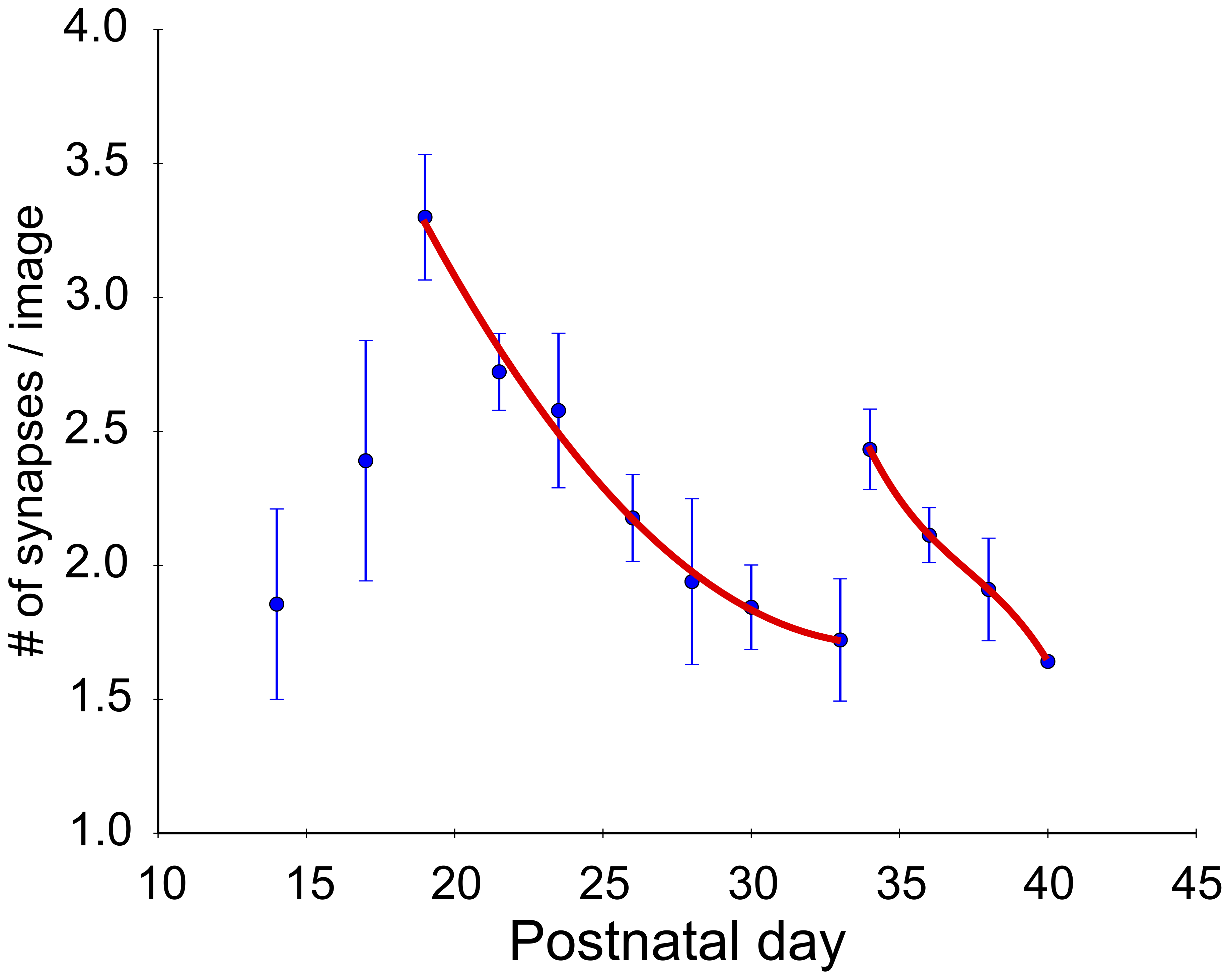

Supplement: S6 Fig — (TIFF) [file pcbi.1004347.s007.tiff]

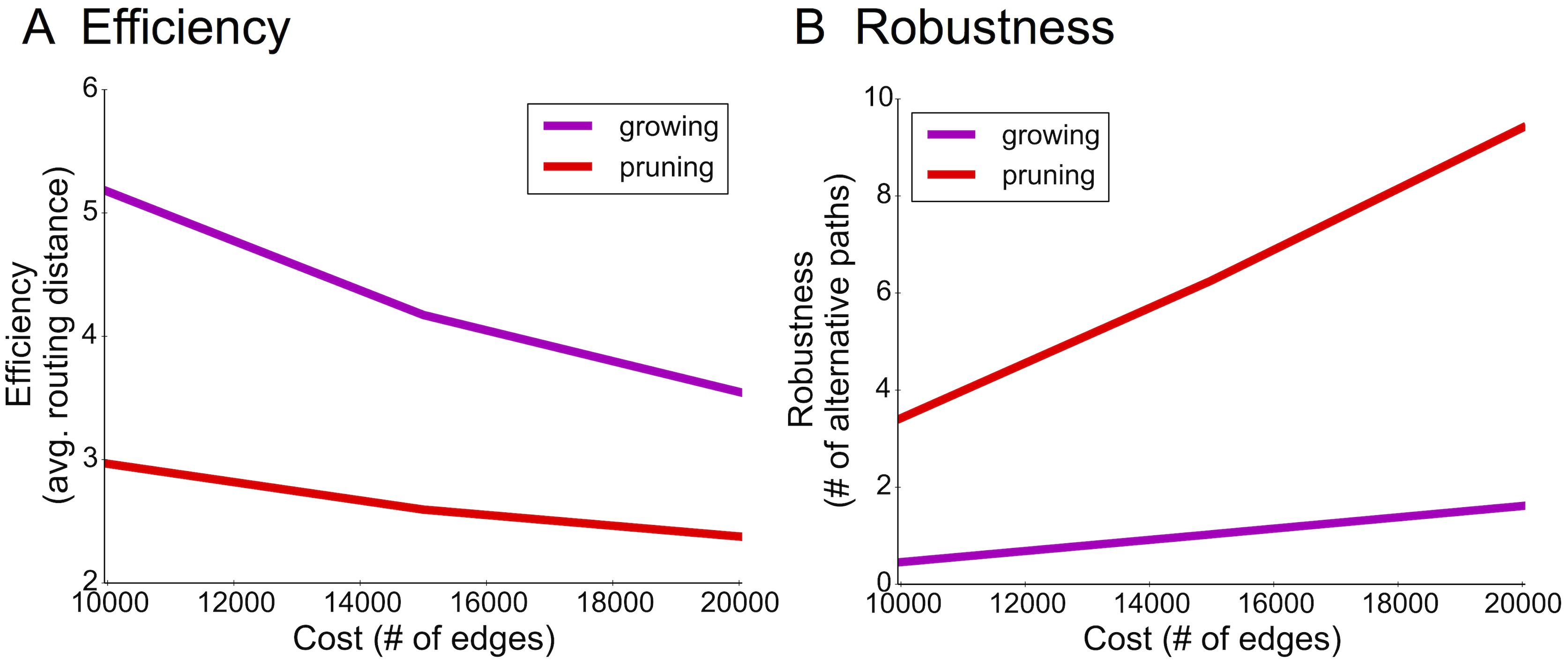

Supplement: S7 Fig — (TIFF) [file pcbi.1004347.s008.tiff]

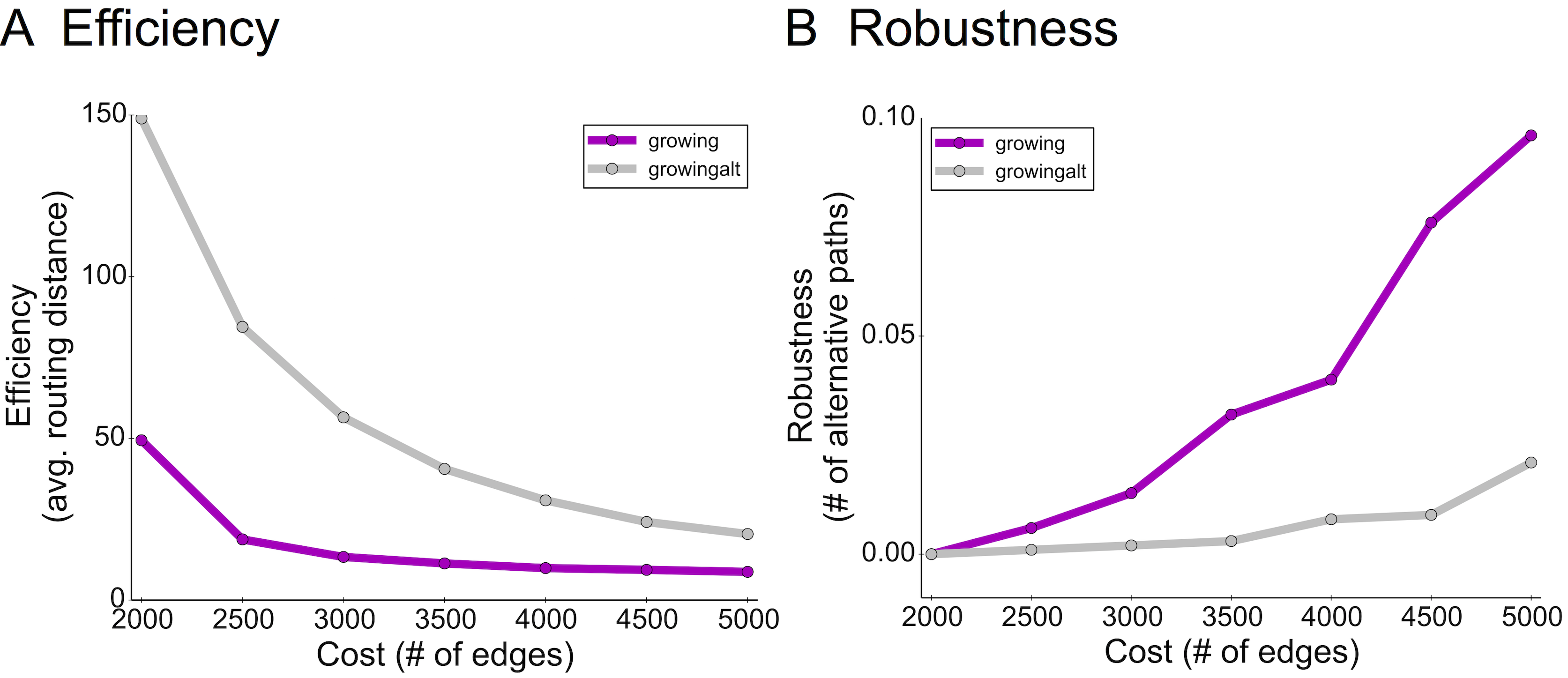

Supplement: S8 Fig — (TIFF) [file pcbi.1004347.s009.tiff]

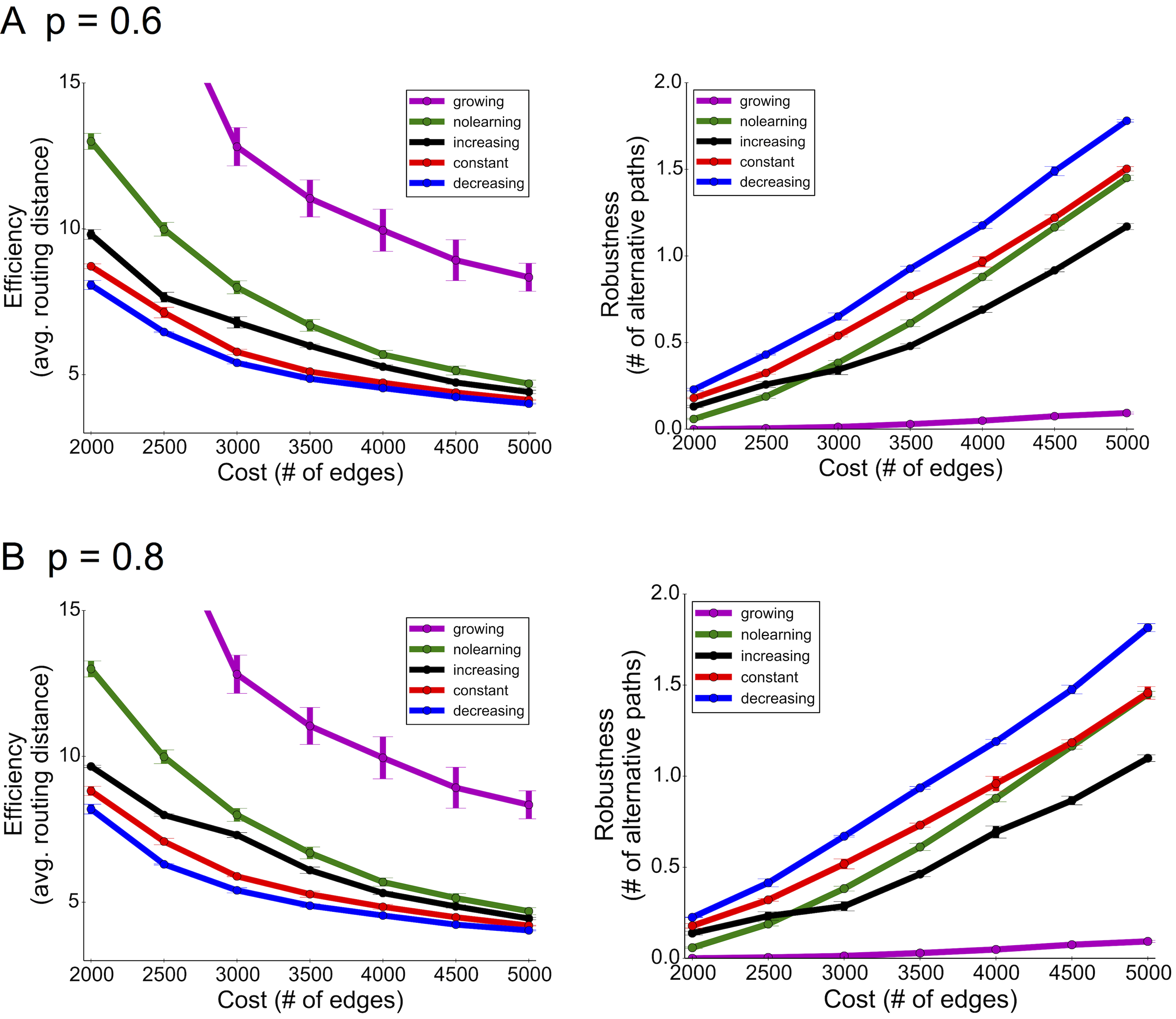

Supplement: S9 Fig — A) Initial density is 60% (i.e. each edge exists independently with probability 0.6. B) Initial density is 80%. (TIFF) [file pcbi.1004347.s010.tiff]

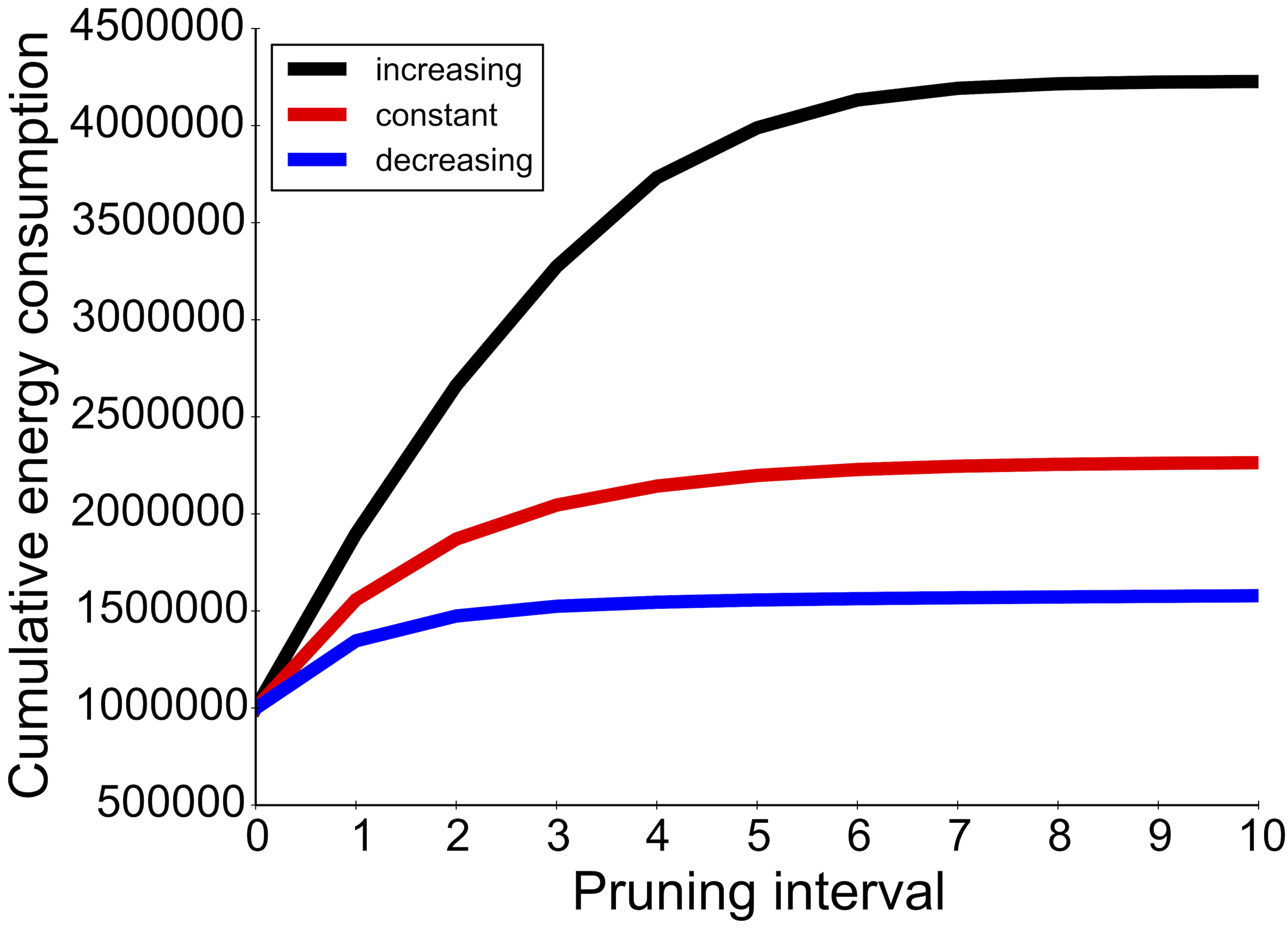

Supplement: S10 Fig — Energy consumption at interval i is the cumulative number of edges present in the network in interval i and all prior intervals. Here, n = 1000 and it is assumed that the network initially starts as a clique. (TIFF) [file pcbi.1004347.s011.tiff]

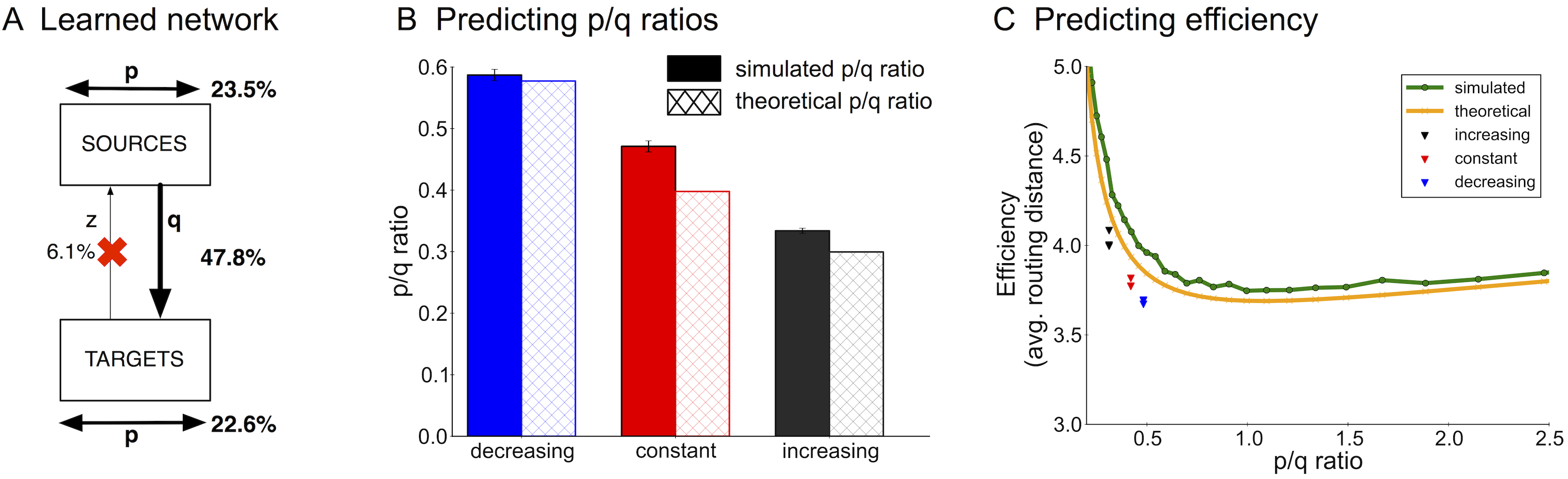

Supplement: S11 Fig — (A) Example edge-distribution using decreasing pruning rates and the 2-patch distribution. (B) Prediction of final network p/q ratio given a pruning rate. Bold bars indicate simulated ratios, and hashed bars indicate analytical predictions. (C) Prediction of source-target efficiency given a p/q ratio. (TIFF) [file pcbi.1004347.s012.tiff]
